# Relating underrepresented genomic DNA patterns and tiRNAs: the rule behind the observation and beyond

**DOI:** 10.1186/1745-6150-5-56

**Published:** 2010-09-22

**Authors:** Miklos Cserzo, Gabor Turu, Peter Varnai, Laszlo Hunyady

**Affiliations:** 1 Department of Physiology, Semmelweis University, Budapest, Tuzolto Street. 37-47. 1094, Hungary, EU

## Abstract

**Background:**

One of the central problems of post-genomic biology is the understanding of regulatory network of genes. Traditionally the problem is approached from the protein-DNA interaction perspective. In recent years various types of noncoding RNAs appeared on the scene as new potent players of the game. The exact role of these molecules in gene expression control is mostly unknown at present, while their importance is generally recognized.

**Results:**

The Human and Mouse genomes have been screened with a statistical model for sequence patterns underrepresented in these genomes, and a subset of motifs, named *spanions*, has been identified. The common portion of the motif lists of the two species is 75% indicating evolutionary conservation of this feature. These motifs are arranged in clusters at close proximity of distinct genetic landmarks: 5' ends of genes, exon side of the exon/intron junctions and 5' ends of 3' UTRs. The length of the clusters is typically in the 20 to 25 bases range. The findings are in agreement with the known C/G bias of promoter regions while access much more sequential information than the simple composition based model.

In the Human genome the recently reported transcription initiation RNAs (tiRNAs) are typically transcribed from these *spanion *clusters according to the presented results. The *spanion *clusters account for 70% of the published tiRNAs. Apparently, the model access the common statistical feature of this new and mostly uncharacterized non-coding RNA class and, in this way, supports the experimental observations with theoretical background.

**Conclusions:**

The presented results seem to support the emerging model of the RNA-driven eukaryotic gene expression control. Beyond that, the model detects *spanion *clusters at genetic positions where no tiRNA counterpart was considered and reported. The GO-term analysis of genes with high concentration of *spanion *clusters in their promoter proximal region indicates involvement in gene regulatory processes. The results of the analysis suggest that the gene regulatory potential of the small non-coding RNAs is grossly underestimated at present.

**Reviewers:**

This article was reviewed by Frank Eisenhaber, Sandor Pongor and Rotem Sorek (nominated by Doron Lancet).

## Background

The regulation of eukaryotic gene expression is one of the central problems in recent biology. The currently ruling view of the process relies on protein - DNA interaction as the initiation step [1]. Transcription factors bind to a distinct point of the chromosome in a cooperative manner and recruit RNA polymerase II and other proteins forming a large transcription initiation complex, and finally make the RNA copy of the gene in question. The first step of the process, i.e. the recognition of the relevant part of the DNA by the transcription factors, drives the gene regulation.

This model is challenged recently by Mattick presenting a coherent and impressive system of arguments why eukaryotes would never work in this way [2,3]. Briefly, the specificity of the protein - DNA interactions is unsuitable to regulate the genetic network at that high level what the collected experimental data suggests. On the other hand, RNA - DNA interaction provides much higher level of specificity and better way of gene expression control. Therefore he concludes: "*it now seems increasingly likely that most of the human genome, and those of other complex organisms, encodes a vast and hitherto hidden layer of regulatory RNAs*."

Since his call for a new paradigm of gene expression control experimental data started to accumulate showing the presence of large population of small non-coding RNAs in various organisms confirming his prediction [4-6]. The most recent one reporting the identification of tiRNAs is particularly interesting [7]. The quasi uniform size and strong positional preference of this ncRNA class towards to the transcription initiation sites suggest a true subtype of closely related fragments. While the applied high throughput techniques are capable detecting these RNA populations and the authors are convinced about their involvement in gene regulation in general the specific biological functions and mechanisms associated with them are still cryptic. The accumulated observations do not form a mature model yet, thus any supporting input is valuable at that stage [8,9].

We implemented a statistical model on simple but very unusual principles and tested it on Human and Mouse genomes, which supports the emerging new model of eukaryotic gene regulation. In short we are looking for underrepresented patterns in complete genomes. The principal characteristic of gene regulation is its efficiency and specificity. In this context specificity means a code hidden in the DNA restricted to certain dedicated genetic locations. These coding fragments are biologically functional at their native place but malfunctional or even harmful at random locations therefore these fragments are under evolutionary pressure not to be present elsewhere but at their correct place. Evidently, restriction in location also means restriction in numbers, likewise in-frame stop codons restricted in numbers and locations in coding regions, according to their biological function. The presented statistical model capitalizes on this property of the patterns.

The predictions of our model are highly correlated with the published tiRNA dataset. The model captures the core principles of tiRNAs providing the theory for the experimental finding and extending the search to other, previously not considered genetic locations.

## Results and discussion

### Identifying underrepresented motifs in genomes

The statistical model is based on head-spacer-tail DNA motifs (see fig. S1; additional file 1). Both of the head and tail units are 6 bases in length allowing two mismatches on each. The model considers all the possible 2560 by 2560 (~6.5 million) head-tail combinations (see the *Methods *for the details). The occurrences of each motif were counted in the Human genome as the function of the spacer length between the units. This procedure provided 6.5 million frequency profiles for the head-tail pairs. In the next step the profiles were analyzed selecting the ones with the shape of a predominantly flat line with a single spike downwards (see fig. S2 and S3; additional file 1). The corresponding head-tail pairs are present in approximately same frequency in the genome regardless of the spacer length but at one critical spacer length where the observed frequency is much lower. About 3% of possible patterns exhibit this feature of reduction in numbers at critical spacer lengths; we call them *spanions *after the ancient Greek for 'rare'. The *spanions *are associated with a weight value proportional with the spike on their frequency profile in "the deeper the spike - the higher the weight" fashion (spike index). See additional 1 for more details of spike index and the list of *spanions *for Human and Mouse as additional files 2 and 3 (*spanion *libraries).

### Scanning Human genome for *spanion *clusters

**Figure 1:**
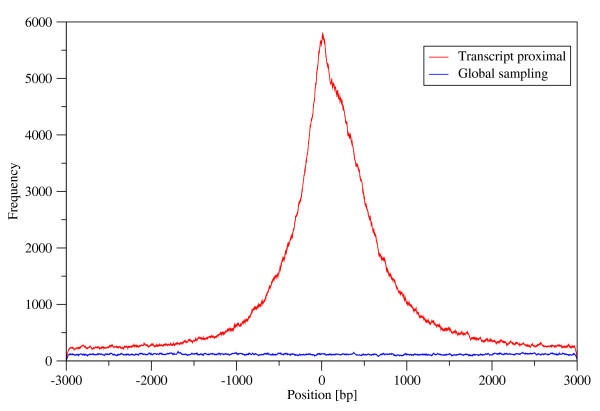
**Distribution of *spanion *clusters around the transcription start sites of genes**. The plot demonstrates the location preference of ***spanion ***clusters inside the fragments of the two Human databases. The transcript initiation site positions of the transcript proximal fragments are aligned to the zero position of the scale according to the ENSEMBL annotations. The position axis scale has no importance in the case of global sampling database. The ***spanion ***cluster content of the two databases (the area under the curves) is 0.29% and 5.2% relative to the full size of the databases respectively. The global sampling dataset represents the average ***spanion ***concentration of the genome including intergenic regions and repetitive sequences as well. Mouse data are not shown.

The *spanion *library, the collection of heads and tails in critical spacer distance, is used in a scoring procedure where the spike index accumulated at matching positions of the sequence in question (see table S4, fig. S7 and S8; additional file 1). This raw scoring profile then processed filtering the high scoring segments of overlapping *spanion *patterns characterized by deep spikes in their frequency profile (see Methods for the details). These segments containing high concentration of *spanion *motifs - *spanion *clusters - are typically 18 - 26 bases in length, G/C rich and highly concentrated in the close proximity of transcription start sites of genes in Human (fig. 1 and fig. S9; additional file 1). This is in agreement with the observation that the Human genome is generally G/C poor, consequently, underrepresented motifs are G/C rich [10]. The flat baseline of the fig. 1 is the result of the scoring procedure performed on the reference database. This database represents the mixture of sense and antisense fragments of every possible genetic context in a manner of their genome wide presence and it is directly comparable in size with the transcript proximal database. In this way the baseline of fig. 1 indicates the average *spanion *cluster content of the genome. The *spanion *cluster lists of the Human transcript proximal database and its Mouse equivalent are presented as additional files 4 and 5.

**Figure 2:**
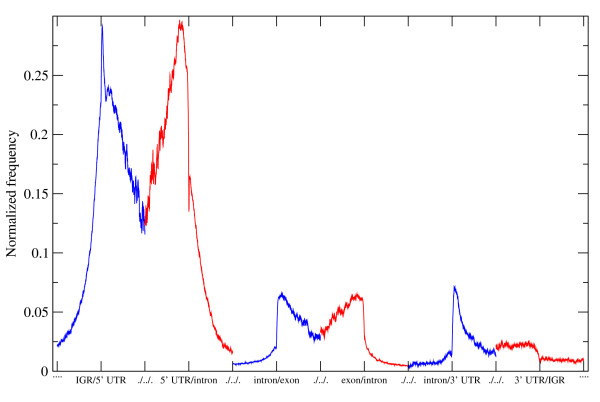
**Normalized distribution of *spanion *clusters around distinct genetic landmarks**. Human fragments are aligned at six key genetic landmarks and the preferred location of ***spanion ***clusters are presented on the plot. The individual distributions arranged into a single plot concatenating them in head to tail fashion representing a hypothetical gene. The original distributions are represented by alternating colours: ***IGR/5' UTR (blue); 5' UTR/intron (red); intron/exon (blue); exon/intron (red); intron/3' UTR (blue) and 3' UTR/IGR (red)***. In each case the point of the alignment is the junction of the two genetic features in question. The possible number of hits decreases with the distance from the selected landmark as fragments are considered only up to the next genetic borderline or 1 kb distance at most. Therefore the raw counts normalised with the maximal number of possible hits at each given position relative to the aligned landmark.

The scoring procedure was applied to the Human gene set too and resulted in 534,002 hits genome wide. The identified hits were mapped to the Human chromosomal assembly and analyzed. The genome was divided into segments along selected genetic landmarks resulting 5' and 3' UTRs with internal exons between them separated by introns respectively. The segment upstream of the 5' UTR is the intergenic region. Due to the large variations in segment sizes the data is presented in normalized form on fig. 2. The presented profile is the location specific *spanion *cluster density of a hypothetical gene with the structure of *intergenic region (IGR) - 5' UTR - intron - exon - intron - 3' UTR - IGR*.

Introns and intergenic regions contain very low number of hits in general but at the ends of the segments with the highest concentration of hits at the very IGR/5' UTR junction. The *spanion *cluster concentration is high all along the 5' UTR segment with positional preference towards to the upstream end. The cluster concentration is much lower in exons and no apparent positional preference observed. In 3' UTRs the cluster concentration is moderate but the upstream end of the segment is strongly preferred.

### The relation of *spanions *and isochors

The large scale compositional inhomogenity of genomic DNA is a well studied subject [11-15]. Segments with characteristic base composition - called isochors - can be identified and their correlation with various genetic features is already established. Most notably the promoter region of eukaryotic genes is rich in 'CG' dinucleotide, and the role of this motif in gene regulation via DNA methylation mechanism has already been confirmed experimentally. The potential effect of this compositional heterogeneity on the *spanion *statistics should be considered.

The first question: if a certain pattern is unfavored for a chemical or physical reason and therefore underrepresented in general, would this bias the statistics? No, the statistical model uses the flat section of the frequency profiles as internal reference. The spike is the relative reduction of the observed frequencies at the critical spacer position versus the average of the neutral ones. Therefore general reduction of the frequencies will not alter the relative differences between the two sections of the frequency profiles.

The second question: can the more complicated statistical model (like *spanion *statistics) access more information than the already known low resolution approach (i.e. CpG island approach)? For the answer consider to following. A few pages of text can hold very complex information which is encoded by the choice and order of the words according to the grammar of the given language. The high level information content of the text has its low level consequences. For example basic statistics, like frequency of single letters or consecutive letters pairs, can discriminate translations to different languages of the same text but can not recognize the difference of two unrelated text of the same language. The low level statistics can not cope with the "similar vocabulary - different context" situation.

The concepts of isochors and CpG islands are defined as genomic regions of certain base compositions. Consequently, they represent low resolution and non context sensitive approaches. To test what level of contextual information can be accessed by the *spanion *statistics the model was tested on numerous variants of real, randomized and partially randomized datasets. These test databases were generated in such a way that low resolution statistics can not make distinction between the real and the faked variants. However, *spanion *statistics can detect the difference between the originals and the derivatives indicating the presence of contextual information which is out of reach of low resolution statistical model. Apparently, large scale compositional heterogeneity is the low level consequence of higher level sequential information which can be accessed by the presented *spanion *statistics.

The detailed results are bulky and may not be informative for the non-specialist reader therefore they are presented in the additional file 1. In brief, *spanion *motifs are present in real datasets representing about 3% of all possible motifs. There are no *spanions *in the set of biologically meaningless sequences (randomized datasets) or repetitive datasets. Partially randomized datasets produce long list of *spanions *with much reduced spike index values. The reduction of the signal is proportional with the reduction of the contextual information content of the database (di- and trinucleotide shuffled databases, mosaic shuffled datasets). There is 75% overlap between Human and Mouse *spanion *lists indicating strong evolutionary conservation of this feature.

### Correlation of *spanion *clusters and experimental data sets

The presented statistical model performs *in silico *screen of completed genomes for underrepresented DNA patterns. Such an *ab initio *approach selects the patterns on an entirely statistical basis and the procedure will not provide direct information about their biological function. The main advantage of such procedure is its generality: no background information required but the sequence itself. This way the result is not sensitive to the possible annotation errors of the database, including the complete lack of annotation in case of unknown motif types.

As the procedure can not reveal the biological role of the identified patterns it is essential to find a close link between the final outcome of the model and direct experimental data demonstrating the biological relevance. In this test neither optimization nor training were preformed on the model for better fit with the reference data. Detected correlations indicate common principles between the compared datasets.

### The link between *spanion *clusters and RNA polymerase II binding sites

**Figure 3:**
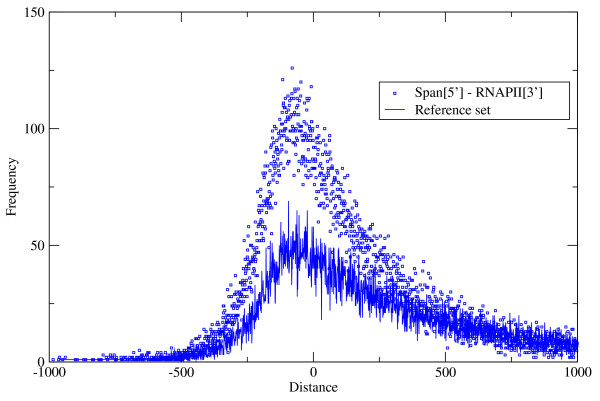
**Co-localization of *spanion *clusters and experimental RNA polymerase II sites**. The distribution of the distance between the upstream end of a ***spanion ***clusters and the downstream end of the closest reported RNA polymerase II sites in upstream position relative to the ***spanion ***cluster [16]. The original measurement has been done on confluent HEK cells. In the reference dataset the local ***spanion ***cluster patterns of the genes were mapped to the chromosomes using the global coordinates of randomly picked different genes. Real dataset: squares, reference set: continuous line. The distribution is asymmetric due to the way the distance is measured. Alternative metrics were also tested and provided similar results (data not shown). The peak around zero in the reference case reflects to the common general preference of the transcript zero position of the two genetic features. Note that the RNA polymerase II sites span a few hundred bases typically while the most common size of the ***spanion ***clusters is around 25 bases. (See Figure 1 and Figure 1 of Sultan et al [16].)

The experimentally determined RNA polymerase II (RNAPII) binding sites of confluent HEK cells was tested against the transcript proximal *spanion *clusters. The published distribution of RNAPII hits relative to the transcription start site is remarkably similar to our fig. 1 (see fig. one of Sultan et al [16]). The positions of the 5' end of *spanion *clusters relative to the 3' end of the upstream closest RNAPII segments were calculated. The distribution of values is presented on fig. 3. The distribution shows strong relative positional preference of the two datasets with maximum at around -80, i.e. *spanion *clusters are typically mapped to the 3' ends of the RNAPII segments. In total 73.5% of the RNAPII segments overlap with at least one *spanion *cluster (see Table 1).

**Table 1:** Co-localization of *spanion *clusters with experimental data (number of reported segments, full length of the segments, number of overlapping segments, length of the overlapping portion of the two sets)

Data	No. of segments	Full length	Hitting segments	Common length
RNAPII	9,710	2,264,991 bp	7,132	842,394bp

tiRNA	1,749	36,328 bp	1,189	22,530 bp

As the overall positional preference of the *spanion *clusters and RNAPII sites is very similar overlap may happen by pure chance. Consider the followings for an analogy: Two persons take the same commuting train every day. They both tend to use the middle of the train as the most convenient choice. The conductor takes note of the two seats occupied by these persons. From the records the distribution of the seat distances can be obtained. It appears the two people tend to sit close to each other. This could be the straight consequence of their independent personal choice or their seat preference could depend on each other: when one of them sits down the other tries to find a nearby place. To distinguish the two possibilities a reference set generated from the original dataset. The seat number records of person "A" is shuffled and associated with the original records of person "B" and this new set is used as the reference distribution of seat distances. In this way the seat choice of "A" on a particular day is compared to the choice "B" on a different day. If they follow their independent preferences the two distributions should be very similar. Whereas, if they actively seek the company of the other they more often travel on nearby places than the distribution of the reference set suggests.

Analogously, for reference the local *spanion *cluster pattern around the 5' end of the genes were mapped to the chromosomal assemblies using the 5' end global position of a randomly picked different gene. This procedure does not alter the overall positional preference of the *spanion *clusters presented on fig. 1 and the original length distribution of the clusters is also maintained while the overlap of the two genetic features drops down to 43.3%. The shape of the curve visibly changes too. This drastic reduction of the co-localization relative to the real case is a clear indication that pure coincidence can not explain the link between the two datasets but it is most likely functional.

The RNAPII dataset of Sultan et al obtained by chromatin immunoprecipitation and subsequent sequencing therefore it is the snapshot of the transcription initiation process itself [16]. The reported segments typically span several hundreds of bases that are at least an order of magnitude longer than the typical length of the *spanion *clusters. Therefore the common portion of the two sets is only 37% of the full length of the RNAPII fragments. The various genetic elements responsible for the expression control of genes are expected to accumulate in the RNAPII hit fragments therefore they most likely contain sub-fragments of diverse functionality in a mosaic like fashion. The biased sensitivity of the statistical model towards to certain types of control elements could explain the moderate *spanion *coverage of the RNAPII sites.

### Relation of *spanion *clusters and tiRNAs

**Figure 4:**
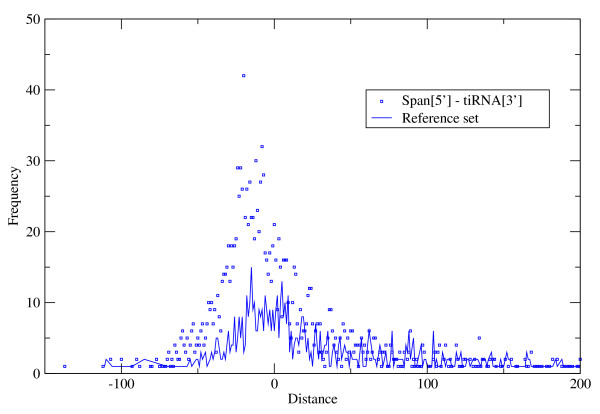
**Co-localisation of *spanion *clusters and experimentally detected tiRNAs**. The distribution of the distances between the upstream end of a ***spanion ***cluster and the downstream end of the closest reported tiRNA hit in upstream position relative to the ***spanion ***cluster [7]. The real dataset is marked by squares; the reference set (continuous line) was generated as in the case of the RNA polymerase II dataset (see above). The typical size of the tiRNAs and the ***spanion ***clusters is directly comparable: 20 - 25 bases. The peak at -20 indicates that typically the 5' ends of the two kinds of segments are only a couple of bases apart.

The overlap of *spanion *clusters and Human tiRNA hits were also tested [7]. Taft et al recently reported this new class of short non-coding RNAs with elevated G/C content associated with the transcription initiation sites of genes. The overlap between RNAPII binding sites and tiRNAs is also reported. This basic description resembles that of *spanion *clusters, and the overlap was measured using the same technique as in case of RNAPII, and the reference set was also generated similarly. The result of the test is presented on fig. 4 and indicates the strong link of the two sets relative to the randomly generated reference case. 68% of the tiRNAs map to overlapping positions with *spanion *clusters the coverage of the overlap is 62% (see Table 1). As per segment and per base ratios are comparable the coverage per segment is close to perfect.

The dataset of Taft et al contains only genome-wide unique segments; therefore the sources of the reported tiRNAs are the corresponding genomic *spanion *clusters. About 30% of the listed tiRNAs overlap with each other. This suggests that the end points of the tiRNA segments are ambiguous or that the applied experimental protocol cause partial degradation of the native tiRNAs during the procedure.

The tiRNA data of Taft et al is originated from deep sequencing experiment of Human THP-1 cells [7]. This technique provides single nucleotide level precision in fragment identification. The size of the dataset (2312 fragments) is sufficiently large for statistical analysis. The overlap of tiRNAs and *spanion *clusters is very high as demonstrated in fig. 4, indicating that tiRNAs are transcribed form their *spanion *cluster counterpart. Practically, the scoring procedure is a potent predictor of tiRNAs.

While the two sets are clearly related there is about 250 times more *spanion *clusters detected relative to the reported number of tiRNAs. We provide plausible explanations as follows:

Firstly, the gene expression pattern strongly depends on the type and state of the cell. Therefore a single experiment can only provide a small subset of all possible markers of gene activity. In contrast, for the statistical model all the potential players of the game are accessible without restriction.

Secondly, Taft et al reports that "*tiRNAs are generally, although not exclusively, associated with highly expressed transcripts*" [7]. On the other hand, the detected concentration of tiRNAs is low. Possibly they pick the most abundant tiRNAs present in high enough concentrations to be detectable under the given experimental conditions and technical limitations of the applied protocol. There well could be a large pool of tiRNAs present in the system at concentrations under the detection limit.

As the third aspect, one should keep in mind that the statistical model has its inherent weaknesses and limitations. The model certainly produces false positive and false negative predictions at a rate which is difficult to estimate presently. For example, the 32% of tiRNAs with no overlap with *spanion *clusters can not be considered as false negatives. The quasi uniform size of tiRNAs does not guarantee uniform functionality of them and the statistical model may have different success rate in prediction of different classes of short RNAs. The selective nature of the statistical model is also apparent in the case of small ncRNA set of Borel et al. It is obtained by size-fractioned RNA extract analysis on DNA tiling arrays covering the ENCODE region of the Human genome [4]. The size of these fragments is in the range of 20 to 50 bases and the reported hits clearly overlap with the *spanion *clusters, however, only 10% of the small ncRNAs have common segment with at least one *spanion *cluster and the extent of the coverage is 7% (data not shown).

For the fourth, and most interesting, aspect is that the genome may contain several types of RNAs with different biological functionality, which are similar in size and statistical characteristics but unrelated otherwise. In this case the *spanion *cluster population contains different subclasses and tiRNAs are only one of them. This exciting possibility is supported by the accumulation of *spanions *clusters at the exon side of the exon-intron boundary, which suggest a function that goes beyond tiRNAs and transciprtion initiation. Note, that Taft et al published only that subset of small non-coding RNAs, which are present in a single copy genome wide.

The correlation between *spanion *clusters and miRNAs have been also tested. Mature miRNAs do not overlap with the *spanion *clusters in statistically significant manner, however, 5' ends of miRNA genes show slight concentration of *spanion *clusters. Due to the relatively low number of miRNA genes the confidence level of this correlation is moderate (data not shown).

It is impossible to estimate the real impact of these four factors in terms of the number of genomic loci and they may well account for the observed 250 times difference in set sizes. It seems plausible to say that the experimentally detected set of the tiRNAs is only the tip of the iceberg. In the real situation they are much more common and tiRNAs are only one class of the several short non-coding RNA types waiting to be discovered.

### What is the biological role?

The apparent strong link between tiRNAs and *spanion *clusters, unfortunately, does not help us to understand the functionality as the biological function of tiRNAs itself poorly understood presently. In a follow up paper the original authors name the aborted transcription events as plausible mechanism for the biogenesis of tiRNAs [9]. However, they are much less confident about the biological function. They list several plausible alternatives, including the possibility that tiRNAs are functionless byproducts. The finding that tiRNAs are built up from a distinct subset of patterns, i.e. *spanions*, makes the 'functionless byproduct' alternative much less likely.

The *spanion *cluster content of exons and 3' UTR regions are considerable as it is presented on fig. 2, therefore these sections are also potential source or target of short RNA molecules. These RNA fragments could be released from the spliced out sections after further processing (i.e. controlled partial degradation). These "gene activity indicators" will recognize their reverse complement counterparts in the mRNAs of other genes. The resulting double stranded structure could trigger RNA interference like mechanisms influencing the expression state of those genes. In this model the small non-coding RNA part provides the specificity of the recognition and tag its target via dsRNA formation.

Numerous examples have been identified in the preliminary results of a more complex analysis where a *spanion *cluster located in an alternative exon of a gene appears in the 5' UTR region of a different gene in reverse complement orientation. *Spanion *clusters present in multiple copies at different regions of different genes define a complex network of potential interactions with high gene regulatory capacity. This possibility was not considered by Taft et al as they concentrate only on fragments present in single copy genome wide and in the close proximity of transcription start sites of genes.

**Figure 5:**
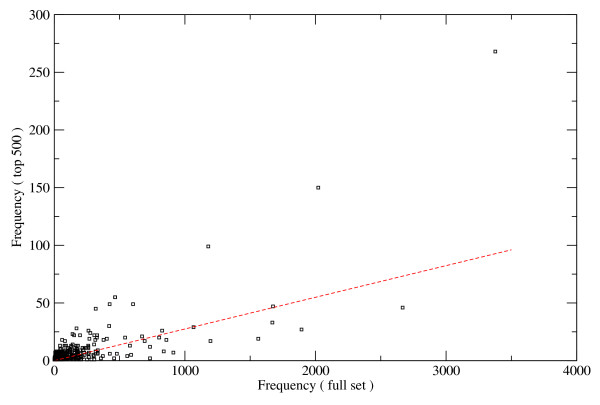
**The relation of *spanion *cluster concentration and GO terms**. The protein coding Human genes were ranked according to the ***spaninon ***cluster content of the 6 kb transcript proximal region. The frequency of the GO terms associated with the 500 top scoring genes was obtained and compared with the GO term frequencies of the full set. In random case one would expect values proportional with the ratio of the sizes of the two sets. This ratio is indicated by dashed line on the plot. Points above the line correspond to GO terms overrepresented in the short listed set of high ***spanion ***cluster content genes. The 25 most frequent GO terms are listed in Table 2.

The transcript proximal regions of protein coding genes were ranked according to their *spanion *cluster content in the hope of additional information about the potential functionality of them. The GO-terms associated with the 500 topmost genes on the list were extracted and the most frequent ones are presented on Table 2[17]. For reference the frequencies of GO terms of the full list were used as presented on fig. 5. In random case the expected frequencies are proportional with the ratio of the short listed set size relative to total number of genes. Interestingly the overrepresented GO terms are strongly associated with gene regulation confirming the likely involvement of *spanion *clusters in these processes.

**Table 2:** The 25 most frequent GO terms associated with the 500 genes richest in *spanion *clusters in their 6 kb transcript proximal region

Freq	GO code	GO term
268	GO:0006355	regulation of transcription, DNA-dependent

150	GO:0045449	regulation of transcription

99	GO:0007275	multicellular organismal development

55	GO:0050826	response to freezing

55	GO:0042309	Homoiothermy

49	GO:0045944	positive regulation of transcription from RNA polymerase II promoter

49	GO:0030154	cell differentiation

47	GO:0007186	G-protein coupled receptor protein signaling pathway

46	GO:0007165	signal transduction

45	GO:0000122	negative regulation of transcription from RNA polymerase II promoter

33	GO:0008152	metabolic process

30	GO:0007399	nervous system development

29	GO:0007155	cell adhesion

28	GO:0009887	organ morphogenesis

27	GO:0006468	protein amino acid phosphorylation

26	GO:0007156	homophilic cell adhesion

26	GO:0006412	Translation

24	GO:0006260	DNA replication

23	GO:0007417	central nervous system development

22	GO:0045941	positive regulation of transcription

22	GO:0008284	positive regulation of cell proliferation

22	GO:0007420	brain development

22	GO:0006357	regulation of transcription from RNA polymerase II promoter

21	GO:0007049	cell cycle

20	GO:0016311	Dephosphorylation

## Conclusions

For the two curve pairs of figs. 3 and 4 an attempt have been made to quantify the difference of shapes relative to the references respectively in terms of statistical significance. The chi-square goodness-of-fit tests resulted 0 probabilities for the null hypothesis, i.e. random chance can not explain the observed overlap of the two set pairs.

Rejection of the null hypothesis, the pure accident, also means that the alternative hypothesis stands. The statistical model captures fundamental aspects of eukaryotic genomes, that is: - 1) reveals a set of segments present only at distinct genetic locations therefore in limited numbers; - 2) this restriction in numbers and locations is closely related to the biological function of these segments; - 3) this functionality is closely linked to the process of transcription and - 4) acting via mechanisms involving small non-coding RNAs. In essence the model provides the list of fragments with distinguished statistical properties, which are likely involved in a new level of gene expression control mechanism acting on the majority of genes.

## Methods

### Databases

The annotation data - 5' and 3' ends, exon/intron boundaries - of known genes were downloaded from the ENSEMBL site via the BioMART interface for the Human and Mouse genomes (version 46, Aug 2007) [18,19]. From the chromosomal assemblies two sets were collected around the reported 5' ends of each gene taking 6 Kb long segments symmetrically resulting 21,342 Human and 21,865 Mouse fragments. These two sets are referred to as transcript proximal databases. The chromosomal assemblies were sampled at every 130 Kb and at each positions a 6 Kb long fragment was picked (global sampling database). The size of this set (21,960) is directly comparable with the transcript proximal one.

From the human genome assembly the gene set database was extracted adding 2 Kb flanking regions relative to the reported 5' and 3' ends of genes.

The list of experimentally determined RNA polymerase II binding sites of confluent HEK cells was extracted from the supplementary data of Sultan et al [16]. The small non-coding RNA hit lists of the HeLaS3, HepG2 and GM06990 confluent cells were extracted from the supplementary data of Borel et al [4]. The tiRNA data of Human THP-1 cells were extracted from the supplementary data of Taft et al [7].

For calculations on more reference datasets see the additional file 1.

### The statistical model

The unit of our model is 6 consecutive bases of DNA (see fig. S1; additional file 1). The next level is the mask that ignores 2 bases out of 6 so a particular unit can have 10 different masks. The four unmasked bases of the unit make 256 variants for each masking patterns. Masking is not allowed at the first position of the unit (certain fragments would be counted twice in consecutive sliding windows otherwise). The final level is the motif: a pair of masked units (head and tail) with a spacer between them in the range from 0 to 52 bases. The sequence of the spacer is irrelevant. The sequence and the masking pattern of the head and the tail are not related - all the possible 2560 by 2560 (~ 6.5 million) variants are considered covering the entire motif space.

A few variants of the above model have been tested: units of 5 bases with one mask, units of 6 with one mask and units of 6 with one and two masks hybrid. The one mask variants were less sensitive and the hybrid did not improve the performance while made the model more complicated.

Generally speaking, shorter units reduce the resolution of the model reducing the number of considered variants. While longer units result in model with very high number of variants. This creates unreasonably high memory demand for the computation and unfavourable for the quality of the statistics as reduces the observed frequency of the individual motifs. This later is especially critical for the analysis of small genomes (like *C. Elegans *~100 Mb). There is no easy way for further improvement of the model with alternative unit size or masking as we have to keep a delicate balance between the number of considered motifs and the observed number of the individual motif types even in the case of small genomes. See the additional file 1 for further technical details of the model.

### The frequency scan and the frequency profile

This model was tested against the various datasets defined above and in the additional file 1 and the raw frequencies of all the possible motifs were counted as the function of the spacer length varying in the 0 to 52 bases range. The resulting raw frequency profiles were normalized to fit into the [0, 1] interval so they can be compared directly:

(1) N=(R−Min)/(Max−Min)

Were *R *and *N *are the raw and the normalised value respectively, while *Min *and *Max *are the minimal and the maximal raw count value of the individual profile.

### Selection of motifs

The normalised values of the individual frequency profiles were sorted in increasing order (*L_1 _*to *L_53_*). The list elements then scanned for *i *satisfying the condition:

(2) $$L_{i}\leq \left(L_{2}+0.2\right)<L_{i+1}$$

In case of *i *< 41 the frequency profile did not pass the filter. The remaining frequency profiles were tested in the second step and accepted if the following logical expression is true:

(3) $$0.4<L_{2}/L_{i}$$

For the selected frequency profiles the average (*Avg*) were calculated using the corresponding raw count values of the *L_2 _*to *L_i _*list elements and the *ln(Avg/Min) *values were tabulated with the head and tail motif (*spanion*). We refer to *ln(Avg/Min) *value as 'spike index' in the text.

In this procedure the frequencies at the neutral spacer distances are utilised as internal standards for the estimation of the random frequency of a given motif. The inherent inhomogenity of the genome prevents the direct calculation of such an estimate without the variable spacer model. The frequency at the critical spacer distance is compared to this reference providing the measures of relative under representation in this way.

The actual selection of 3 parameters (at least 41 points in the 0.2 wide belt and the distance between the lowest and the next point) detects no *spainons *in the randomly generated dataset (see the additional file 1). The flowchart of the filtering procedure is presented on the fig. S3; additional file 1. The critical spacer distance of the profile was also recorded and utilised in the scorning procedure. In practice, the critical spacer distance is 0 for all the *spanions *motifs (i.e. direct head-tail contact) - see the additional file 1 for the details.

### Scoring procedure

The input sequences were scanned applying the sliding window technique, the *spanion *hits were recorded, and the 12 bases of the head and the tail of each *spanion *received the corresponding spike index value. Any actual fragment of 12 bases can receive 100 hits at most, if all of its possible motifs are present on the list of *spanions*. Plotting the score along the sequence defines the scoring profile. Considering the adjacent overlapping segments the score can accumulate as high as several hundreds per bases. This raw scoring profile was analysed in terms of a simple filtering rule set. Only peaks higher than a given limit considered ('top' filter, see fig. S8; additional file 1). The width of the peak is measured at a second, somewhat lower limit as the baseline of the peak ('base' filter). If the peak is sufficiently wide it is considered as a true hit ('wide peak' filter). Alternatively, tandem of peaks can pass the filter, if they are wider than the minimal width ('narrow peak' filter) and the distance in the sequence between them is reasonably small ('gap' filter).

One should keep in mind that the rare character of *spanions *is only apparent on the relative scale compared to their estimated frequencies. In practice the numbers of the individual *spanion *motifs in the entire Human or Mouse genomes are typically in the several ten thousands to few hundred thousands range. Consequently, the statistical model will produce large number of hits with low statistical significance. The main aim of the filtering procedure is the reduction of this noise via discrimination between weakly and strongly significant predictions. In our model the statistical significance is proportional with the signal strength of the scoring profile. Therefore, filtering the segments characterised by high and wide peaks on the scoring profile selects the most significant hits. These *spanion *clusters are short continuous sequence fragments with high concentration of overlapping *spanion *motifs of the statistical model. See table S4; additional file 1 for an example. It is also worth to note the following; while *spanion *clusters are building up from *spanion *motifs mostly this does not guarantee that a particular cluster appears only once genome wide. The reverse is also stands: genome wide unique fragments are not necessarily *spanion *clusters.

The possible combinations of the filter settings were tested systematically on the transcript proximal dataset (fig. 1). The setting that produced the highest peak at the transcription start site, relative to the two side regions of the curve, was used in the calculations. This procedure optimizes the signal to noise ratio via the elimination of the weak hits and keeping the most confident ones.

This rule set is purely empirical and performs equally well for Human and Mouse sequences. The optimal values of the filter parameters are: 'top' = 90; 'base' = 30; 'wide peak' = 25; 'narrow peak' = 13 and 'gap' = 10. The implementation of the scoring algorithm with the *spanion *library is available upon request in Linux binary format.

## Authors' contributions

MC contributed by the basic idea, initial calculations and the draft of the manuscript, GT, PV and LH contributed in equal manner by critical improvements and extensions of the applied methods, analysis, interpretation and presentation of data and the final manuscript preparation. LH also supervised the project. All authors read and approved the final manuscript.

## Reviewers' comments

### Reviewers report 1 - Frank Eisenhaber

*"The authors describe a method to determine underrepresented pairwise combinations of hexanucleotides with spacers of differing length between them (with up to two mismatches for each motif half) and find that up to 3% of these pairs have drastic reductions of occurrences at specific spacer lengths. These motifs are called spanions. Spanions are reported to be clustered and to occur at genomic locations that are correlated with (i) some isochors and notably CpG islands, (ii) RNAPII binding sites, and (iii) locations of tiRNAs and several other small RNAs*.

Whereas the work brings up important observations and successfully connects them with previous knowledge, the manuscript would benefit from considering the following issues:

1) Is there any motivation why the authors analyze motifs of the type hexanucleotide-spacer-hexanucleotide and restrict the mismatches to two on each side? Why not one mismatch or penta-/heptanucleotides, why is the spacer introduced?"

Authors' response: The presented statistical model is only the most potent one amongst the tested variants. A short paragraph in the 'Methods' section introduced in the revised version about the general considerations of the model construction and the experience with the less successful candidates. The use of the spacer is also described briefly (see Methods: 'The statistical model' and 'Selection of motifs').

"2) The work would greatly benefit from presenting detailed data for a single representative spanion and a single representative non-spanion so that the reader gets a feeling what kind of data does this analysis produce (to be inserted at page 3 bottom/page 4 top)."

Authors' response: The full list of Human and Mouse *spanions *are submitted as additional files 2 and 3. The Table S4 (additional file 1) provides an example for *spanion *motifs. These changes are also requested by the other two referees.

"3) The section "spanions and isochors" finally does not clarify what is the relationship between them. How many spanions are "near" CpG islands or isochors (what is the distance relationship)? Generally, the authors are scarce with exact numbers; instead, the trends in the data are described with words here and throughout the text. Maybe, it would be good to summarize in a table all absolute numbers of spanions, RNAP sites, tiRNAs, etc. and how many of those overlap sequentially."

Authors' response: The text of the section is altered on several places and answers the question in its present form. A new table have been added listing the correlation data of RNAPII/tiRNA segments and the *spanion *clusters. The data in the CpG islands vs. *spanion *clusters relation is available in the additional file 1 section. In our opinion it would be slightly confusing to present the correlation of *spanion *clusters with experimental results and a prediction method (CpG islands) in the same table.

"4) The author should provide a algorithmic definition of what is a spanion cluster with all parameters in the main text (top of page 5)."

Authors' response: The detailed description of the scoring algorithm and the filtering procedure is included in the relevant 'Methods' section and referenced at the main text. We introduced a Supplementary Table and a Supplementary Figure presenting the concept in a visual way. (These changes were also requested by Rotem Sorek.)

"5) The language of the MS would benefit from polishing. Some sentences are incomplete (e.g., 2nd sentence of last paragraph of page 7)."

Authors' response: The MS is modified on several points according to the comments of the referees (including that particular sentence) and hopefully the most confusing parts are corrected in the recent version.

"6) The abstract would benefit from including all conclusions and some of the most important numerical results in this MS; at present, it is too verbose without the interesting pieces of information provided in this work."

Authors' response: The 'Abstract' now includes the most important conclusions of the work in an explicit form.

### Reviewers report 2 - Sandor Pongor

*"The work of Cserzo and associates presents a statistical analysis of rare sequence motifs in the human genome. They find that the rare sequence motives, termed spanions cluster in the vicinity of RNA pol II and tiRNA binding sites. These interesting observations shed light to a relatively less known property of genomic sequences which certainly deserves systematic analysis. The central concept of this work is the role of rare motifs. In my opinion the statistical analysis of rare motifs is a particularly important topic. According to the working hypothesis of this work, rare motifs carry specific functions. The naive reader expects that rare motifs should coincide with promoters and other protein binding sites. In contrast, the present work shows that rare motifs cluster around various other binding sites, notably tiRNA and RNA pol II binding sites, which points to the currently perhaps underestimated - role of the latter in regulatory events*.

1) The present analysis is based on direct enumeration of bipartie motives consisting of two hexanucleotides connected with a spacer of varying length. It is not clear to me whether the bipartite motives are chosen because of their similarity of transcription factor binding sites or because of their "enumerability". Namely, direct enumeration of DNA motives is a computationallly hard problem which can be apparently solved on this particular subset. While addressing this question in the manuscript the authors may also comment on the percentage of the motif space they analyze so that the reader can get an impression about the generality of the conclusions."

Authors' response: Indeed, our original intention was to identify cooperative transcription factor binding sites at fixed distance in the sequence. Our model could not find those but picked *spanions *instead. Frank Eisenhaber also queried the details of the model; please see our reply to his first point above.

"2) The authors may consider showing a few representative examples of the avoided motifs, motif clusters and give more detailes on how the motif clusters are defined. Also some of the conclusions (e.g.) could be supported by more statistical details given as supplementary information. For instance, a list of spanion clusters for the human and mouse genomes could be given as an appendix."

Authors' response: The *spanion *libraries as well as the *spanion *clusters in the transcript proximal region of Human and Mouse genomes are submitted as Supplementary files as requested. The details of the scoring procedure also illustrated with a new Supplementary Table and Supplementary Figure.

"3) Finally, some of the conclusions I found particularly interesting are not mentioned in the present version of the abstract. The abstract would definitely benefit from a thorough brush-up, according to the guidelines of the Journal."

Authors' response: The abstract is modified as requested.

### Reviewers report 3 - Rotem Sorek

"In this paper Cserzo et al. devised an algorithm to scan the human and mouse genome for underrepresented sequences, which they called spanions. They further found that these spanions are overrepresented in proximity to TSS of genes. The authors infer their results as if spanions are functionally connected to tiRNAs and polII binding sites; however, this is far from convincing, as these features are co-localized to TSSs, where spanions also co-localize. As described below, this is a major flaw of this manuscript as currently written, and more analyses are needed to establish the spanion-tiRNA overlap theory."

Authors' response: It appears that the referee missed a few crucial points of the paper perhaps due to the insufficient depth of explanation. We improved the text in reflection to his comments in the hope he reconsiders his first judgment (see the responses below).

*"Major issues:*

Once the authors showed that spanions are enriched in TSS (and even more bothering: according to the Methods, the filters were set so that spanions **will be **enriched in TSS) it is an expected and a trivial result that spanions will be enriched within tiRNAs and polII binding sites as these two are enriched in TSSs. The authors themselves mention that "the overlap between RNAPII binding sites and tiRNAs is also reported (by Taft et al.)". The authors also stated that tiRNAs were mapped only to unique genomic sequences, which bias their appearances in spanions (those tiRNAs that are not located in unique genomic sequences were not reported by Taft et al.)."

Authors' response: We agree with the concerns of the referee regarding the filtering procedure. However, the filter settings were obtained on the basis of the statistical significance of the individual predictions. The procedure separates weak signals and strong ones detecting - but not generating - enrichment of *spanion *clusters as highly significant hits around the TSSs. Generally speaking, post processing the output of any statistical model - i.e. ranking the predictions according to the signal strength and setting minimal confidence level requirement - is a widely accepted practice. We modified the 'Scoring procedure' section in the 'Methods' to avoid the confusion and emphasize the importance of the filtering.

The referee is right about that the correlation of RNAPII binding sites, tiRNAs and *spanion *clusters is inevitable at a certain extent as all the three genomic features concentrated around the TSSs. The only question is whether the observed correlation exceeds that certain random extent or not. Therefore the correlations of the experiments and prediction were contrasted with the correlations of experiments vs. random reference. The experimental data sets on Fig 3 and 4 result visibly different distributions with the predicted *spanion *clusters relative to the corresponding reference set. This visual proof is expressed in numbers by the results of the chi square goodness-of-fit test (see 'Conclusions'). After all, the observed correlations of RNAPII sites and tiRNAs with the *spanion *clusters are well beyond the level one can expect by pure coincidence.

"Based on this, the title should be changed to exclude mentioning tiRNAs, as the authors have absolutely no evidence that these spanions are connected to the phenomenon of tiRNAs."

Authors' response: The evidence is presented on Figure 4 as the difference of the real set and reference set.

"I also don't understand the reference set selection. If a spanion cluster occurrence is a rare event in the genome, isn't it expectable not to see the same event re-occurring in a random gene and in a specific distance from polII binding site/tiRNA?"

Authors' response: The generation of the reference set is one of the crucial points of the paper. It is absolutely essential to understand this step for the correct interpretation of the results demonstrating the close link between the *spanion *clusters and the experimental observations. Accordingly, detailed explanation via an example is introduced in the 'The link between *spanion *clusters and RNA polymerase II binding Sites' section.

"The statistical model:

Most known motifs have a single, possibly degenerate, consecutive pattern (often represented by a position weight matrix) and the motifs you are searching have a unique pattern of fixed head-spacer-fixed tail pattern. Please explain the logic behind the model; why did you decide working with such a complex pattern? Why did you select to work with 6 consecutive bases of DNA in the head and tail? Why masking of 2 bases was determined? What is the logic behind the decision to work with fixed flanking sequences and a variable spacer size? Please denote if these preferences are based on any empirical computational results or a biological principal."

Authors' response: Please see our answer to Frank Eisenhaber's first question and Sandor Pongor's first remark.

"It is also important to note in the paper, and not only in the Sup. Information part, that the vast majority of spanions (~95%) had a spacer size = 0 (Sup. Table1-2), which means that they are actually a consecutive 12-bp degenerate motif (a 'normal' motif), and not a bipartite motif recognized by its distinct head and tail separated by unimportant sequence."

Authors' response: Done (see Methods: 'Selection of motifs', last paragraph).

*"Results and discussion - the relation of spanions and isochors*.

This part should be shortened and moved to the end of the discussion. It was hard to understand the connection of this part to the spanions phenomenon until reading, in the next page (page 5), that spanions are GC rich and enriched in TSS, similarly to CpG islands."

Authors' response: According to our experience the conceptual difference of CpG islands and *spanion *clusters is a major issue for the potential readers. We changed the order of these two paragraphs as suggested but we prefer the lengthy and detailed version for better understanding.

"Results and discussion - Scanning Human genome for spanion clusters

*The concept of spanion clusters repeats throughout the paper and is not clear*.

Please give an example for a spanion cluster and its creation from different spanions, preferably as a Sup. Figure."

Authors' response: The explanation is in the second paragraph of the new version of 'Scoring procedure'. Briefly, *spanions *are the motifs of the statistical model while *spanion *clusters are sequence fragments with high *spanion *content. It is also presented via an example (Tab. S4; additional file 1).

"Figure 1* and *2*: The impression one gets from these figures is that spanions are enriched in TSS. However, the authors are not showing whether the **rarity **of these sequences might contribute to the positional bias. Therefore, I suggest that the authors would add as another control to the analysis, data of non-spanions from your initial analysis, that is, sequences that are represented as expected or overrepresented in the genome. It would be more interesting if the enrichment in TSS is specific to spanions and not to non-spanions. Also, in figure *2, *it would be useful to add the normalized frequency of randon intergenic regions that are far from genes."*

Authors' response: Figure 1 shows the *spanion *cluster part of the transcript proximal region. As this subset is overrepresented around the TSSs its complementary set is necessarily underrepresented. We can not see the benefit presenting this trivial fact on the plot.

"Figure 4

*Regarding *figure 4*: since spanions and tiRNAs are approxiamately in the same size (whereas spanions are much smaller the polII binding sites) it would be much comprehensible if the distance between these elements would be presented as the distance between the 5' edges of both element types. Actually, if tiRNAs are ~18 bp long, and the distance peak is around -20 bp between spanion start and tiRNA end, then both elements should start in the same position."*

Authors' response: The 5' ends of the segments are indeed close to each other in number of cases. We made this explicit in the legend of the figure. However, we prefer to use the same metric as in case of the RNAPII were the *spanion *clusters tend to accumulate 80 bases upstream of the 3' end of the segments and the chosen metric suits for that. In our opinion the identical metric together with the notes in the legends makes the two figures more comparable.

"Relation of spanion clusters and tiRNAs

If tiRNAs are presumably functional RNAs and the authors want to show that spanions are related to functional RNAs, it would be convincing if the tiRNAs are enriched within conserved (in human-mouse) spanions."

Authors' response: The main aim of this step is to establish the relation between the statistical model and experimental evidences. The referee is right about that it would be a stronger argument if we could access and analyze tiRNA data for mouse. We are eager to do so as soon as the data will be available. Till that we have to rely on Human data.

"It would be also nice to see if tiRNAs are enriched within spanions of higher spike index."

Authors' response: Only the fragments which are concentrating high spike index value *spanions *can pass the filtering procedure. Please see our response concerning the filtering procedure above.

*"At the moment I am not convinced that tiRNAs are overlapping with the very large list of transcript proximal spanions (> 200K according to Sup. *Table 1) *just because the two groups co-localize in the TSS of genes."*

Authors' response: According to our results the observed co-localization of the two sets exceeds the level of the correlation what would be expected by pure chance. Please see our response concerning the generation of the reference set few comments above and the related improved version of the text.

"Page 8 - the paragraph starting with "For the fourth". The authors hypothesize that spanions are enriched in some other small RNA groups, in addition to tiRNAs. My first guess was that spanions would overlap with many miRNAs as these are short (~22 bp) similarly to spanion clusters, noncoding, and many of them are found in single copies in the genomes and transcribed mainly by polII (spanions are near polII binding sites). However, the authors states in the sup. information that according to their finidings "even if the detected spanion clusters are related to microRNAs this link is rather weak.". I believe this negative result should appear in the paper and not in the sup. information as many readers would think of it."

Authors' response: The moderate correlation of miRNA genes and spanion clusters is mentioned as suggested in the 'Relation of *spanion *clusters and tiRNAs' section.

*"Minor issues:*

Please supply the full list of spanions/spanion clusters, preferably with their spike index, as a supplementary material."

Authors' response: The Human and the Mouse *spanion *libraries for the scoring procedure are included as Supplementary Files.

*Page 2, last line: change 'what' to 'that' (a typo)*.

Authors' response: We rephrased the sentence.

"Page 3, line 7 from the end of page: You are explaining the calculation of 2560 possibilities in the methods part but here this number is confusing since 4^6 = 4096. Please explain the calculation here (4^4*10 = 2560) or refer to Methods."

Authors' response: The reference to the Methods section included.

"Figure 2* - the usage of two colors here is little confusing. If the different colors are used only to emphasize each separate region, then please mention it in the figure legend. It is also worth mentioning in the text that the lack of continuity in the frequency of spanions in the border between an intron and coding/noncoding exon and the fact that intron edges are very poor in spanions may be caused by the fact that intron edges are characterized by clear overrepresented splicing signals (5' and 3' splice sites, polypyrimidine tract) which cannot be spanions by definitions. This bias contradicts the example given by the in the fourth explanation for the large number of spanions (upper part of page 8)."*

Authors' response: The legend to Figure 2 is modified accordingly to the suggestion. The referee is right about that exon/intron edges are poor of *spanion *clusters but they appear at the exonic side close to the junction. The text is corrected accordingly at the referred place.

"Page 4, paragraph starting with the second question: the context of the question itself is not clear (what is the complicated statistical model and what is the low resolution approach here?) and so does the example given as an answer. Maybe you should start the paragraph by defining that isochors determination is a low resolution approach is, as mentioned in the next paragraph, and you are suggesting here the 'spanion statistics' concept which is a more complicated model."

Authors' response: The paragraph modified accordingly to the request.

"Page 5, line 13 - "spanions are G/C rich" - it is worth mentioning that this is an expected result as the genome is GC poor (41% GC according to the Lander et al. Nature 2001 Initial sequencing and analysis of the human genome) so we expect underrepresented motifs to be GC rich."

Authors' response: The remark and the reference are included as suggested.

"Page 6, without the notion that RNAPII segments span several hundred bases (that appear afterwards) in comparison to the ~25 bp of a spanion, the following sentence is unclear: "The distribution shows strong relative positional preference of the two datasets with maximum at around -80, i.e. spanion clusters are typically mapped to the 3' ends of the RNAPII segments." - since a short RNAPII segment might be downstream to a spanion if their distance is negative."

Authors' response: The correction mentioning the size difference of the two sets is introduced in the legend to Figure 3.

"Page 8: "Numerous examples have been identified where a spanion cluster located in an alternative exon of a gene appears in the 5' UTR region of a different gene in reverse complement orientation." -please add at least one reference."

Authors' response: This is the preliminary results of our more complex analysis. We made it explicit at the referred place in the text.

"Page 10, line 6 for the end - change 'point' to 'points'."

Authors' response: Done.

*"Sup. *Figure 4 *- The authors suggest in the sup. information (page 3, line 7 from the bottom) that the spanions are underrepresented genome wide, but these otherwise rare fragments accumulate at the proximity of start sites of genes. It will be more convincing that the peak here is caused by spanions in TSS, if the global sampling set will be divided into those that are in TSS and those not in TSS."*

Authors' response: Fig. S4 (additional file 1) presents the distribution of the spike indexes calculated from two human database sections and also suggests the preference of *spanion *motifs towards to the TSS segments in an indirect way. This, indeed, could be supported with the suggested division of the global sampling set. However, Fig. 1 and 2 answers this question in the most direct way presenting the specific genetic locations where the *spanion *motifs are concentrated. Therefore, in our opinion, the suggested change would mean very little improvement relative to the original version while would require repeated calculations on the two parts of the database.

## Supplementary Material

Additional file 1 **Supplementary information**. The file contains further technical details of the model and the results of excessive testing on various reference datasets. The file also contains 9 additional Figures and 4 Tables. Click here for file

Additional file 2 **Human *spanion *library**. The list of ***spanion ***motifs with their spike index values calculated from the global sampling database of the Human genome. The included motifs are those with zero critical spacer and spike index bigger than 0.75. File format: UNIX style plain text, readable by the 'WordPad' application under 'Windows'. Click here for file

Additional file 3 **Mouse *spanion *library**. The mouse equivalent of Human ***spanion ***library. Click here for file

Additional file 4 **List of *spanion *clusters in the Human transcript proximal dataset**. FASTA format list of ***spanion ***clusters in the Human transcript proximal database. File format: UNIX style plain text, readable by the 'WordPad' application under 'Windows'. Click here for file

Additional file 5 **List of *spanion *clusters in the Mouse transcript proximal dataset**. The mouse equivalent of Human *spanion *cluster list. Click here for file
