# Supplementary material for: Relating underrepresented genomic DNA patterns and tiRNAs: the rule behind the observation and beyond
Source: Biol Direct. 2010 Sep 22;5:56. doi: 10.1186/1745-6150-5-56 (PMC3583238; doi:10.1186/1745-6150-5-56)
Supplement: Additional file 1 — Supplementary information. The file contains further technical details of the model and the results of excessive testing on various reference datasets. The file also contains 9 additional Figures and 4 Tables. [file 1745-6150-5-56-S1.PDF]

Supplementary information to 'Relating underrepresented genomic DNA patterns and tiRNAs: the rule behind the observation and beyond'

By Miklós Cserző, Gábor Turu, Péter Várnai, László Hunyady

Department of Physiology, Semmelweis University, Budapest

Technical details of the statistical model and the characteristics of the underrepresented patterns.

The frequency profile approach

Here we describe and analyse the DNA sequences in terms of a hierarchical statistical model (see Fig. S1 for graphic representation). Our motif is a head and tail pair of fragments separated by a variable length spacer. Considering the length of the units of our model, the size of the complete genome and the restricted genomic alphabet of the 4 bases, the head or the tail alone can not be sufficiently specific for any gene regulatory purpose as the number of model units is only 2560. However, in the case of functionally linked head and tail the number of variants is 2560 by 2560 ~ 6.5 million providing more than 3 orders of magnitude better specificity. In our model 'link' would mean that a particular head and tail pair with a critical spacer between them fulfils a specific biological functionality, but the same head-tail couple is not effective at any other relative sequential positions.

The search for functionally important head-spacer-tail combinations is based on the frequency profile analysis of the motifs. This is the observed number of a certain head and tail motif in the database as the function of the spacer length between them. The raw counts were normalised according to Equation 1. The examples of the relevant profile types are presented on Figure S2. Here we examined the spacer in the range from 0 – direct head-tail contact – up to 52 bases. One cannot expect functional link at very large separation of the units and the chosen spacer range proved to be suitable according to the results presented below.

For random sequence set random frequency profiles are expected. If there is no link between the head and the tail, the spacers are equivalent, and, as a consequence, the frequency does not vary significantly with the varying spacer length. Therefore the normalised values of a frequency profile are scattered quasi-symmetrically over the whole interval (see panel "A" of Fig. S2).

In case of "B", "C" and "D" type profiles the outscoring point relative to the flat baseline identifies the critical spacer of the particular head – tail combination.

Reference dataset

We have analyzed the motif statistics on a randomised database first, which was a collection of 22,000 fragments, 6Kb long each. Eukaryotic genomes show elevated C/G content around the transcript start, whereas more distant regions are A/T rich. These sequences were generated randomly reflecting to this position dependent compositional bias, and mimicking a transcription start site at the exact middle in this respect. The C/G content is around 40% at both ends of the fragments which increases gradually to 60% towards to the middle, i.e. the simulated transcription start site. The actual C/G content profile is compiled from the transcript proximal database. Furthermore, the individual sequences were not related to each other or to any real DNA fragment of an organism.

The frequency profile analysis of this dataset mostly results “A” type curves. The other 3 types are missing from the result set of curves.

Human and Mouse datasets

The frequency profiles of Human transcript proximal segments and the global sampling database, as well as their Mouse counterparts were also analysed. The results of the statistics for these segments differ drastically from the random data. The most apparent difference is the very high number of panel “B” type profiles of Figure S2. This is the consequence of the repetitive sequence content of the genome.

The second difference between the random and real sequences is the considerable number of panel “C” type profiles. These represent motifs of far less frequency at a critical spacer distance than any other relative positions of the head and the tail. Although these motifs are much less frequent than type “B” ones, we have found plenty of such examples in the Human and Mouse genomes, as well. These are not present in the randomized control sample, and it definitely can not be caused by repetitive sequences (see the discussion later). Our presented research is focused on these motifs as a likely indication for a yet unknown biological functionality.

Selection of motif list

The frequency profiles were subjected to a filtering procedure. The flowchart of the procedure is presented on Figure S3. This step aims to rescue the panel ”D” type profiles (Fig. S2) which are the combinations of “B” and “C” types. The filtering procedure selects motifs represented by a special frequency profile: a single outscoring minimum below a predominantly flat line. The filter passing combinations of head, critical spacer length and tail was termed as *spanion*.

In case of Human and Mouse transcript proximal databases 231,534 and 277,734 *spanions* passed the filter, respectively, that is 3% - 4% of all the possible motifs. The filter is highly selective as only 1 weakly significant motif can pass it applying the filter to the randomised dataset.

Characteristics of the short-listed motifs

The most important characteristics of these *spanions* are presented in Table S1 and Table S2. Considering the position of the spike, the vast majority of the

spanions had a critical spacer length = 0. The number of *spanions* drops sharply with the increasing critical spacer length reaching the flat, very low baseline at spacer 5 and 10 for the two kinds of databases respectively. This confirms that the maximal spacer length = 52 is more than enough for the analysis. Even ignoring a few outscoring points of repetitive origin per frequency profiles is acceptable with respect to the quality of the statistics.

Comparing the *spanion* lists of the two species the overlap is rather high at spacer = 0, however there is no overlap at the section of longer spacers, i.e. the same head-spacer-tail combinations share similar statistical characteristics in the genome of the two studied organisms. Assuming that the *spanions* with longer critical spacers are consequences of suboptimal filtering and statistical deviation the large overlap at spacer = 0 indicates evolutionary conservation. At this point we refer to the familiar “important things are conserved” logic of biology and as the *spanion* lists show conservation, the property that they are reflecting is most likely important.

The spike index

The importance of the various *spanions* is not equivalent, since the corresponding frequency profiles show considerable variety in terms of the size of the downward spike relative to their flat section. The measure of this property is the spike index. The distributions of the spike indexes are presented in Figure S4 for the Human transcript proximal and the global sampling databases. Both distributions show the presence of two populations: a group of relatively small spikes at the low end and more characteristic, deeper ones at the upper end of the scale. The boundary between the two populations is at spike index = 0.75. One may have the impression that the high end population shifted further upwards in case of global sampling database, while large portions of the low end one has been lost. Comparing the *spanion* lists behind the two halves of the distributions this guess is confirmed.

A high spike index means that the *spanion* is highly underrepresented. The shift to the higher value means that the *spanion* is even less common in the global sampling database. Note, that the transcript proximal database contains the sense orientation of 5’ end of a gene in the very middle of the 6 Kb fragment according to the annotation, whereas global sampling database represents the mixture of sense and antisense fragments of every possible genetic context in a manner of their genome wide presence. Consequently, the result indicates that the *spanions* are underrepresented genome wide, but these otherwise rare fragments accumulate at the proximity of start sites of genes. Furthermore, the coherency of the two sets also indicates that the calculation is not sensitive to the possible annotation errors of the database in terms of the transcript zero points.

The results of the Mouse genome are in perfect agreement with the Human genome ones. Comparing the high end spike index populations of motifs between the Human and the Mouse they share 75% of *spanions* representing a very strong evolutionary conservation, while the two lists contains only 2.5% and 2.9% of all the possible motif types, respectively (see Table S2). The

conservation of *spanion* lists is qualitatively different than the conservation of sequences, and should not be confused as the former one is far more indicative.
As the *spanions* of high spike index population are correlated more strongly with the transcription start of the genes these narrowed down lists were used as motif libraries in the scoring procedure. (Additional files 2 and 3.)

Analysis of datasets with partially preserved information content

Real DNA sequences exhibit a complex framework of sequential correlation of the bases. This non-random character results in the occurrence of various types of clusters with characteristic biased composition (isochors) [1-3]. A series of test calculations was carried out to evaluate the contribution of the various short range sequence correlations to the occurrence of *spanions*. Random datasets were generated so that low level statistics can not make distinction between the originals and its derivatives. If the *spanion* statistics can detect the difference that indicates the capability of this method to access short range sequential information out of reach for low level representation.
First, the sequences of the promoter proximal databases were shuffled using the 'uShuffle' tool of Jiang et al. [4]. The resulting databases retain the exact di- and trinucleotide composition of the originals, respectively. The algorithm is capable of producing shuffled sequences with preserved composition at the tetra- or pentanucleotide level, or even beyond that, but in practice the shuffled sequences in these cases tend to preserve longer fragments in intact form than the desired level. Therefore only the dinucleotide and trinucleotide shuffled datasets were used as references.
Our statistical pipeline detected a long list of *spanions* in the dinucleotide shuffled dataset; in fact, the list is much longer than the one obtained for the transcript proximal database (see Tab. S3) and the overlapping portion of the two lists is also considerable. The strong preference for critical spacer length = 0 was also found. Apparently, the rather limited short range information content reflected in the dinucleotide composition of the shuffled database defines the strong critical spacer preference of *spanions*. However, in regard to the spike index, the *spanion* list of the shuffled database is only a poor approximation of the real situation (see Fig. S5). Clearly, more information is needed than the dinucleotide composition for the full explanation.
In the second test the trinucleotide shuffle algorithm was applied on the transcript proximal database, which resulted in an even longer list of *spanions* (Tab. S3) and a marginally larger overlap with the original transcript proximal *spanion* list. The spike index distribution changed drastically relative to the dinucleotide shuffled case. Most of the list elements shifted towards zero; while the index improved for the small subset of *spanions* only. Despite of this improvement the spike index distribution is still visibly worse than the one of the original list. This difference indicates that the information content of trinucleotide composition does not provide sufficient explanation for the statistical characteristics of *spanions* in the genome.
Unfortunately, further gradual increment of the information content via the implementation of tetra- or pentanucleotide shuffling is technically impossible.

Alternatively, the mosaic shuffling applied on the transcript proximal database. In this procedure the input sequence is fragmented to units of *n* nucleotide. These mosaics shuffled then to produce the randomised output. The possible residual fraction at the end of the original is appended to the end of the output as it is. This procedure was applied to the transcript proximal database using *n* = 3, 4, 6, 8, 10, 12, 14 and 16. The resulted databases are referred to as mosaic 3, 4, etc. respectively.
This algorithm keeps the local sequential preferences up to the size of the mosaic in a gradually decreasing manner. For example in case of mosaic size of 6 the 5/6-th of the original dinucleotide structure is conserved; 4/6-th of the trinucleotide etc. down to 1/6-th in case of hexanucleotides. The output of the procedure is shuffled beyond recognition relative to the original considering the alignment of a few randomly selected input – output sequence pairs. In this approximation the conservation level of the sequential preferences cannot reach 100%, but can get close to it with the increasing mosaic size.
Consider the results of the spike index distribution of *spanions* of the various datasets shuffled with different mosaic sizes (see Fig. S6 and Tab. S3). Apparently, longer the mosaic unit more the distribution looks like the one of the original transcript proximal database. However, even the mosaic 16 distribution is visibly different relative to the original. However, it is difficult to tell whether this difference is due to the approximate nature of the mosaic model or due to the significance of sequential preferences in that range.
Considering these tests, one should keep in mind that shuffled sequences are not random despite the fact that alignment algorithms can not detect the relation between the originals and the shuffled sequences. Due to their partial information content shuffled sequences are conceptually different and behave differently than random ones in tests for example with respect to the free energy of RNA folding [5].
On the other hand, comparing the results of the dinucleotide, trinucleotide and mosaic 3 shuffled datasets suggests that the system is very sensitive to changes of the short range information content. As our statistical model is designed to be sensitive for short range sequential features this behaviour is expected. In these cases comparable information content result considerably different *spanions* lists and spike index distributions as well. In a further test a second variant of the dinucleotide shuffled version of transcript proximal database was generated and analyzed. The *spanion* lists originating from the two independent copies are similar in size (375,129 and 373,951 *spanions* respectively) but the common portion of the two is only about 80% (304,790 *spanions*).
Apparently, the lists obtained form various databases with different partial information content share a common core as they all overlap largely with the *spanion* list of the transcript proximal database. Therefore these different results are coherent but inaccurate approximations of the real case.
At least these tests demonstrate that the presence of *spanions* in the genomic DNA is caused by complex sequential preferences well beyond the trinucleotide level. The strong preference of the detected *spanions* for critical spacer length = 0 can be explained by sequential preferences at the dinucleotide level. The low

statistical quality of the results on these datasets most likely does not permit further conclusions.

Redundancy of datasets

Human and Mouse genomes contain significant portions of repetitive fragments. This is a major concern for methods looking for overrepresented motifs. However, here we concentrating on underrepresented motifs. A motif in the repetitive portion of the genome occurs in high copy number therefore simply by definition cannot be a *spanion*, i.e. low copy number motif.
To confirm this hypothesis, the repeat-masked version of the Human genome was downloaded from the ENSEMBL site and analysed. High number of *spanions* was detected in this database (293,341), and the spike index distribution was very similar to that obtained from the global sampling database (data not shown). Comparing the two lists there are 167,480 list elements are common, while the high spike index portion of global sampling *spanion* list is 96% common with the *spanion* list of the repeat-masked database.
In addition, the list of repetitive elements was retrieved via the MySQL interface of the ENSEMBL site [6]. From this list a representative subset was selected randomly containing interspersed and tandem repeats longer than 300 bases and the corresponding sequence fragments were retrieved from the database. The size of the resulting set is comparable with the size of the transcript proximal dataset and its randomized derivatives.
This repetitive sample concatenated with the randomized, the di- and the trinucleotide shuffled databases, respectively. Statistical analysis of these datasets resulted in low number of *spanions* with very low spike indexes in the case of the randomized + repetitive sample hybrid, while di- and trinucleotide shuffled datasets joined with the repetitive sample contain approximately the same number of *spanions* as the original datasets, respectively. The spike index distribution is also preserved in similar manner (data not shown). Apparently, the addition of repetitive sequences had no major effect on the *spanion* lists. In fact, the presence of repetitive sequences improves the result as demonstrated for the data of Table S3.

Analysis of DNA sequences

The *spanions* within the DNA sequences can be located and analysed in terms of their genetic context using the obtained *spanion* libraries. For an example of the scoring curve see Figure S7. (Human type-1 angiotensin II receptor, [ENSEMBL:ENSG00000144891]). Generally, the raw curves are noisy therefore a filtering back-end implemented as integral part of the scoring procedure (see *Methods*). A single hit indicates only a moderate level of restriction on the frequency of a *spanion*, whereas accumulation of hits represents higher level of restriction i.e. cluster of *spanions*, suggesting the presence of a highly specific fragment. The applied filter screens the cumulated hits according to their width, height and density selecting the top scoring fragments (see Fig. S8).
The sizes of the individual *spanion* clusters were also examined (see Fig. S9). Clearly, there is a preferred *spanion* cluster size in the range of 18 to 23 bases;

longer continuous hits are much less common. In close inspection longer hits rather appear as tandems of two or more consecutive *spanion* clusters, each of them within the range, forming compact assemblies.

Weakly related genetic features

Although the preferred *spanion* cluster size is remarkably close to the size of microRNAs, especially, if we allow some uncertainty due to the potentially suboptimal scoring procedure, microRNA genes show some, but not pronounced concentration of hits (data not shown). Furthermore, microRNA targets are in the 3' UTRs of genes unlike the presented hits, which are predominantly concentrated in the 5' UTRs. In conclusion, even if the detected *spanion* clusters are related to microRNAs this link is rather weak.

Eukaryotic promoter regions are G/C rich and these fragments are commonly known as 'CpG islands'. The definition of this genetic feature is based on the very simple statistical measure: CG dinucleotide content in a window is estimated from the C/G single nucleotide content and compared with the observed CG dinucleotide count. If C/G content is high and observed vs. expected CG dinucleotide ratio is high than the segment is a CpG island [7]. Recommended window sizes are between 200 and 500 bases. Shorter or longer windows are not practical due to unfavourable false positive and false negative prediction rates respectively. Consequently the shortest window size limits the resolution of the method in the few hundreds of bases range.

Further, the definition is unable to distinguish two fragments as long as their base composition and CG dinucleotide content is identical. Thus the CpG island concept describes the genetic features in a very low resolution in terms of sequence specificity as well. Recently the 'CG clusters' suggested as a more precise alternative instead of the previous definition [8]. This method is based on a slightly different but yet simple statistical measure therefore suffers from similar problems.

For example the 'CpGplot' tool of the EBI site detects CpG islands in high numbers in the position dependent composition biased reference set. Whereas the *spanion* statistics is able to recognise the difference between the real and random sets not finding *spanions* in the later.

Certainly the *spanion* clusters correlate with 'CpG islands' and 'CG clusters' in the sense they both concentrated in the promoter proximal regions of genes. According to the annotation *spanion* clusters accounts for only 35% of the promoter proximal CpG islands in length. Reversing the viewpoint CpG island definition over predicts our *spanion* clusters by 300%. On the other hand, *spanion* clusters are frequent at other genetic locations where CpG islands are not present (like at exon / intron junctions). Therefore the two definitions differ fundamentally with respect to their predictive power: CpG islands are the approximate genetic locations of potential gene regulation activity, while *spanion* clusters are the possible actual fragments directly involved in gene regulation.

A randomly selected subset of the Human transcript proximal database was screened for potential transcription factor binding sites against the JASPAR database via the provided web interface [9]. Despite the relatively high

confidence level of 90% the server predicted potential binding sites rather promiscuously covering about 37% of the fragments in length. In contrast to that only ~31% of the detected *spanion* clusters were overlapped with predicted binding sites in that subset of fragments, indicating that *spanion* clusters are not enriched in transcription factor binding sites.

Supplementary Figure legends

Figure S1 – Graphical representation of the model

The structure of the applied hierarchical model is presented here and the employed terms are illustrated by an example.

Figure S2 - Frequency profile examples

The plot presents basic types of frequency profiles. The averages of the profiles are indicated by the dotted lines. See the text for the details.

Figure S3 – Graphical representation of the filtering

The frequency profiles are piped into a filter that selects the ones with distinctive statistical properties. The consecutive steps of the process are presented here as flowchart.

Figure S4 - Spike index statistics

Distribution of spike index values of the motifs passing the statistical filter described in the *Methods*. Curves present data of the two Human datasets, Mouse equivalents are not shown.

Figure S5 – Spike index distributions of the di- and trinucleotide shuffled databases

From the original transcript proximal database two shuffled derivatives were obtained of preserved di- and trinucleotide compositions. The resulting frequency profiles were screened with the statistical filter providing the *spanion* lists for these two databases. The distribution of spike indexes is presented. The original distribution of the transcript proximal database is also included for comparison.

Figure S6 – Spike index distributions of the mosaic shuffled databases

Spike index distributions of the mosaic shuffled derivatives of the transcript proximal database.

Figure S7 – Representative example of a score curve

A typical example of score curve. The result of the scoring procedure applied on 4 Kb segment of the Human type-1 angiotensin receptor II sequence. The transcript start site is at 0 according to the ENSEMBL annotation. The false positive peaks of the raw scoring curve are removed during the post-processing. The positions of the filter-passing *spanion* cluster hits are indicated as black sections by the arrow above the curve. The unit of the score is the cumulated spike index.

Figure S8 – Filtering of the raw scoring profile

The statistical significance of the *spanion* cluster hits is proportional with the signal strength of the scoring profile. The filtering procedure selects those hits which are characterised by sufficiently high and wide peaks. The first two peaks

of the example do not pass the filter: the first is too small (top-filter), the second is to narrow (base-filter). The third one is a good hit while the fourth one is a twin of high but narrow peaks. As they are reasonably close to each other they also pass the filter (gap-filter).

Figure S9 – Distribution of *spanion* cluster lengths

The plot presents the statistics of *spanion* cluster lengths on the Human transcript proximal database. Mouse data are not shown.

Table S1 - Motifs statistics, transcript proximal databases

	Spanions	Spacer = 0	Spacer > 10
Human	231,533	218,238	90
Mouse	277,733	254,602	138
Common motifs	135,006	133,866	0

Motif statistics of the two transcript proximal databases and the intersection of the two sets presented above. Columns list the total number of *spanions* and subsets according to the headings.

Table S2 - Motifs statistics, global sampling databases

	Spanions	Spacer = 0	Spacer > 5	High spike index
Human	206,094	201,579	62	163,021
Mouse	243,434	235,782	67	188,817
Common motifs	140,383	140,238	0	132,567

Motif statistics of the two global sampling databases (50 Kb densities) and the intersection of the two sets presented above. Columns list the total number of *spanions* and subsets according to the headings.

Table S3 – *Spanion* statistics on various reference databases

Shuffled database	*Spanions*	Overlap
Mosaic 3	189,564	124,173
Mosaic 4	240,167	143,577
Mosaic 6	276,413	153,536
Mosaic 8	292,184	152,096
Mosaic 10	296,372	142,426
Mosaic 12	289,571	128,676
Mosaic 14	276,376	117,972
Mosaic 16	259,756	109,255
Dinucleotide	375,130	174,329
Dinucleotide+repeat	206,832	129,138
Trinucleotide	503,126	172,363
Trinucleotide+repeat	222,250	126,581

Motif statistics of the shuffled derivatives of the Human transcript proximal database is presented above. The columns are the name of the database, the number of detected *spanions* and the common portion of the reference and the

high spike index portion of the original transcript proximal *spanion* list respectively according to the headings.

Table S4 – Example of a *spanion* cluster and the list of its *spanion* motifs

TCCAGCGCCGAGGCGCGCGCAGAG		TCCAGCGCCGAGGCGCGCGCAGAG		TCCAGCGCCGAGGCGCGCGCAGAG	
T..AGCG.C.AG	1.79	A..GCCG.GG.G	0.78	AG..GCG.GCA.	1.25
T..AGCGC..AG	2.00	AGC..CG.GG.G	1.09	AG..GCG.GC.G	0.99
T..AGCGCC.A.	1.98	AG..CCG.GG.G	1.10	A.G.GCG.GC.G	1.05
T..AGCGCCG..	1.19	AG.G.CGA..CG	1.66	AGG..CG.GC.G	1.05
T..AGCGCC..G	1.86	AG.G.CGA.GC.	1.66	GC.C.CG.A.AG	1.50
T..AGCGC.GA.	2.03	AG.G.CGAG.C.	1.72	GC.C.CG.AGA.	1.55
T..AGCG..GAG	1.79	AG.G.CGAGG..	0.97	GC.C.CG.AG.G	1.30
T.CA.CG.C.AG	1.64	AG.G.CGAG..G	1.54	GC.C.CGCA.A.	1.03
T.CA.CGC..AG	1.70	AG.G.CGA.G.G	1.58	GC.C.CGCAG..	1.14
TC.A.CGCC.A.	1.24	AG.G.CG.GGC.	0.86	GC.C.CGCA..G	1.18
T.CA.CGCC.A.	1.66	AG.G.CG.GG.G	0.79	GCG..CG.A.AG	1.17
T.CA.CGCCG..	1.18	C.AG.CGC..GC	1.20	G..CGCG.A.AG	1.26
TC.A.CGCC..G	1.19	C.A.GCG.GC.C	0.85	GC..GCG.A.AG	1.56
T.CA.CGCC..G	1.52	C.AG.CG.GC.C	1.29	GC..GCG.AGA.	1.67
TC.A.CGC.GA.	1.67	C..GGCGC..GC	0.97	GCG..CG.AG.G	0.86
T.CA.CGC.GA.	1.68	C..GGCG.GC.C	1.11	G..CGCG.AG.G	0.98
T.CA.CG..GAG	1.73	A..CGCG..CAG	1.06	GC..GCG.AG.G	1.25
TCC..CG.C.AG	1.42	A.GC.CG..CAG	1.45	GC..GCGCA.A.	1.16
TCC..CGC..AG	1.54	AG.C.CG..CAG	1.46	GC..GCGCA..G	0.99
TCC..CGCC.A.	0.88	AG.C.CGC..AG	1.54	GC..GCGCAG..	1.21
TCC..CGCCG..	0.76	A.GC.CGC..AG	1.71	GC..GCGC..AG	1.26
TCC..CGC.GA.	1.59	A.GC.CGC.CA.	1.12	GC..GCGC.GA.	1.26
T.C.GCG.C.AG	1.48	AG.C.CGC.CA.	1.39	GC..GCGC.G.G	0.76
T.C.GCGC..AG	1.67	A.GC.CGC.C.G	1.25	GC..GCG..GAG	1.01
T.C.GCGCC.A.	1.55	AG.C.CG.G.AG	1.29	G.GC.CG.A.AG	1.49
T.C.GCGCC..G	1.23	A.GC.CG.G.AG	1.34	G.GC.CG.AGA.	1.47
T.C.GCGC.GA.	1.81	A.GC.CG.GCA.	1.14	G.GC.CG.AG.G	1.35
T.C.GCG..GAG	1.68	AG.C.CG.GCA.	1.28	G.GC.CGCA.A.	1.13
A.C.CCGAG.C.	1.35	AG.C.CG.GC.G	0.96	G.GC.CGCA..G	0.93
A.C.CCGAG..G	1.27	A.GC.CG.GC.G	1.01	G.GC.CGCAG..	1.22
A.C.CCGAGG..	1.33	A.G.GCG..CAG	1.48	G.GC.CGC..AG	1.24
A.C.CCG.GGC.	0.98	AGG..CG..CAG	1.57	G.GC.CGC.GA.	1.23
A.C.CCG.GG.G	1.14	AG..GCG..CAG	1.59	G.GC.CGC.G.G	0.98
A.CG.CGA.GC.	1.27	AGG..CGC..AG	1.71	G.GC.CG..GAG	1.12
A..GCCGA.GC.	1.14	A.G.GCGC..AG	1.76	G.G.GCG.A.AG	1.62
A..GCCGAG.C.	1.14	AG..GCGC..AG	1.79	G.G.GCG.AGA.	1.18
AG..CCGAG.C.	1.43	A.G.GCGC.CA.	1.29	G.G.GCG.AG.G	1.18
AG..CCGA.GC.	1.53	AG..GCGC.CA.	1.51	G.G.GCGCA.A.	1.33
A..GCCGAGG..	1.03	AGG..CGC.CA.	1.64	G.G.GCGCA..G	1.20
A..GCCGAG..G	1.14	AG..GCGC.C.G	1.30	G.G.GCGCAG..	1.23
A..GCCGA.G.G	1.19	AGG..CGC.C.G	1.39	G.G.GCGC..AG	1.25
AG..CCGAGG..	1.39	A.G.GCGC.C.G	1.39	G.G.GCGC.GA.	1.16
AG..CCGAG..G	1.43	AGG..CGCG.A.	0.87	G.G.GCG..GAG	0.92
AG..CCGA.G.G	1.59	AGG..CG.G.AG	1.25		
AGC..CGAG..G	1.62	A.G.GCG.G.AG	1.30		
AGC..CGA.G.G	1.67	AG..GCG.G.AG	1.30		
A..GCCG.GGC.	0.75	AGG..CG.GCA.	1.15		
AG..CCG.GGC.	1.01	A.G.GCG.GCA.	1.18		

The *spanion* cluster is in the first lines of the table across the three columns. The *spanion* motifs are listed subsequently with their spike index values.

References

1. Cohen N, Dagan T, Stone L, Graur D: **GC composition of the human genome: in search of isochores.** *Mol Biol Evol* 2005, **22:**1260-1272.
2. Nekrutenko A, Li WH: **Assessment of compositional heterogeneity within and between eukaryotic genomes.** *Genome Res* 2000, **10:**1986-1995.
3. Schmegner C, Hameister H, Vogel W, Assum G: **Isochores and replication time zones: a perfect match.** *Cytogenet Genome Res* 2007, **116:**167-172.
4. Jiang M, Anderson J, Gillespie J, Mayne M: **uShuffle: a useful tool for shuffling biological sequences while preserving the k-let counts.** *BMC Bioinformatics* 2008, **9:**192.
5. Workman C, Krogh A: **No evidence that mRNAs have lower folding free energies than random sequences with the same dinucleotide distribution.** *Nucleic Acids Res* 1999, **27:**4816-4822.
6. Hubbard TJ, Aken BL, Beal K, Ballester B, Caccamo M, Chen Y, Clarke L, Coates G, Cunningham F, Cutts T, et al: **Ensembl 2007.** *Nucleic Acids Res* 2007, **35:**D610-617.
7. Takai D, Jones PA: **Comprehensive analysis of CpG islands in human chromosomes 21 and 22.** *Proc Natl Acad Sci U S A* 2002, **99:**3740-3745.
8. Glass JL, Thompson RF, Khulan B, Figueroa ME, Olivier EN, Oakley EJ, Van Zant G, Bouhassira EE, Melnick A, Golden A, et al: **CG dinucleotide clustering is a species-specific property of the genome.** *Nucleic Acids Res* 2007, **35:**6798-6807.
9. Sandelin A, Alkema W, Engstrom P, Wasserman WW, Lenhard B: **JASPAR: an open-access database for eukaryotic transcription factor binding profiles.** *Nucleic Acids Res* 2004, **32:**D91-94.

Fig. S1

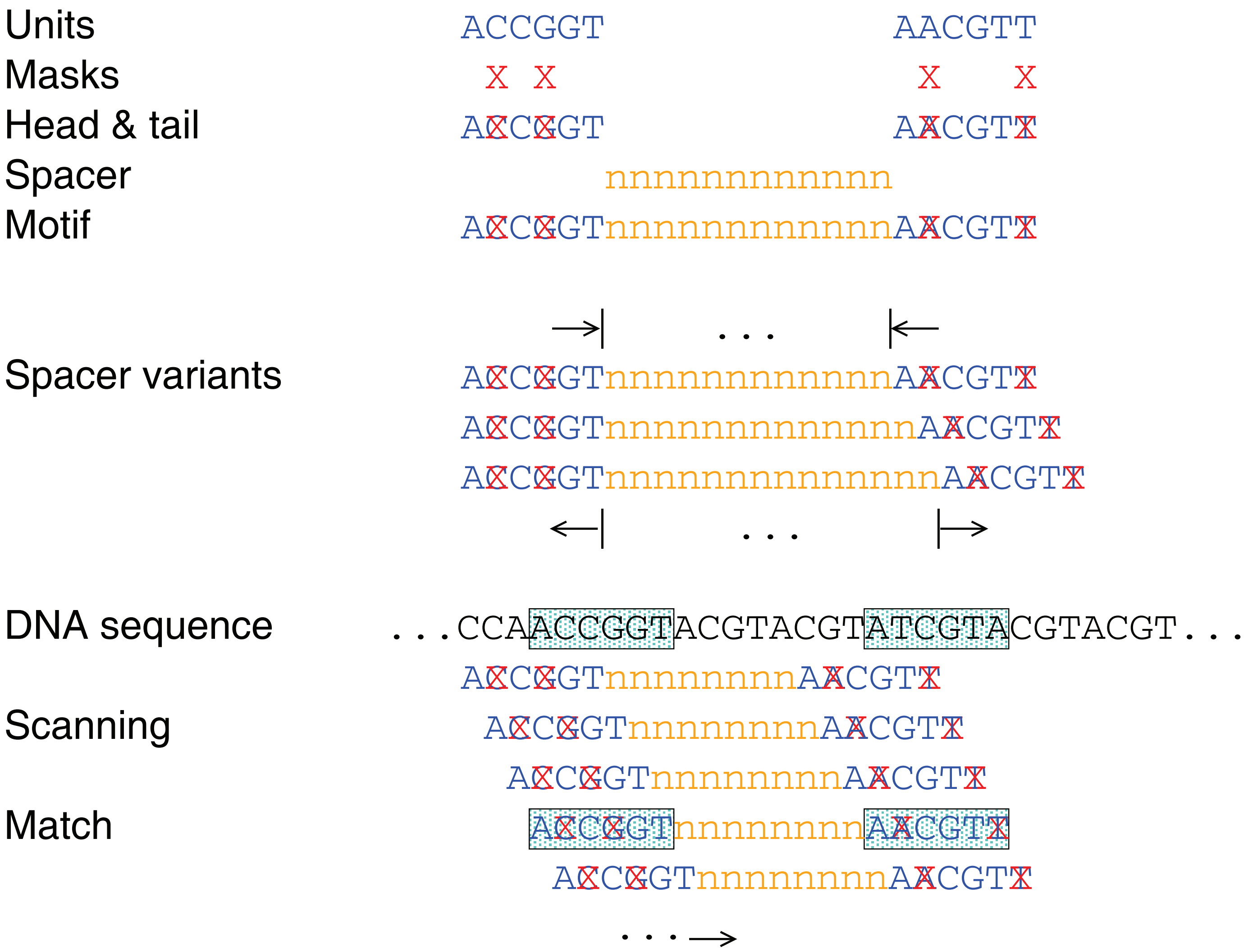
Units
ACCGGT
AACGTT
Masks
Head & tail
Spacer
nnnnnnnnnnnn
Motif
Spacer variants
DNA sequence
. . . CCAACCGGTACGTACGTATCGTACGTACGT . . .
Scanning
Match

Fig. S2

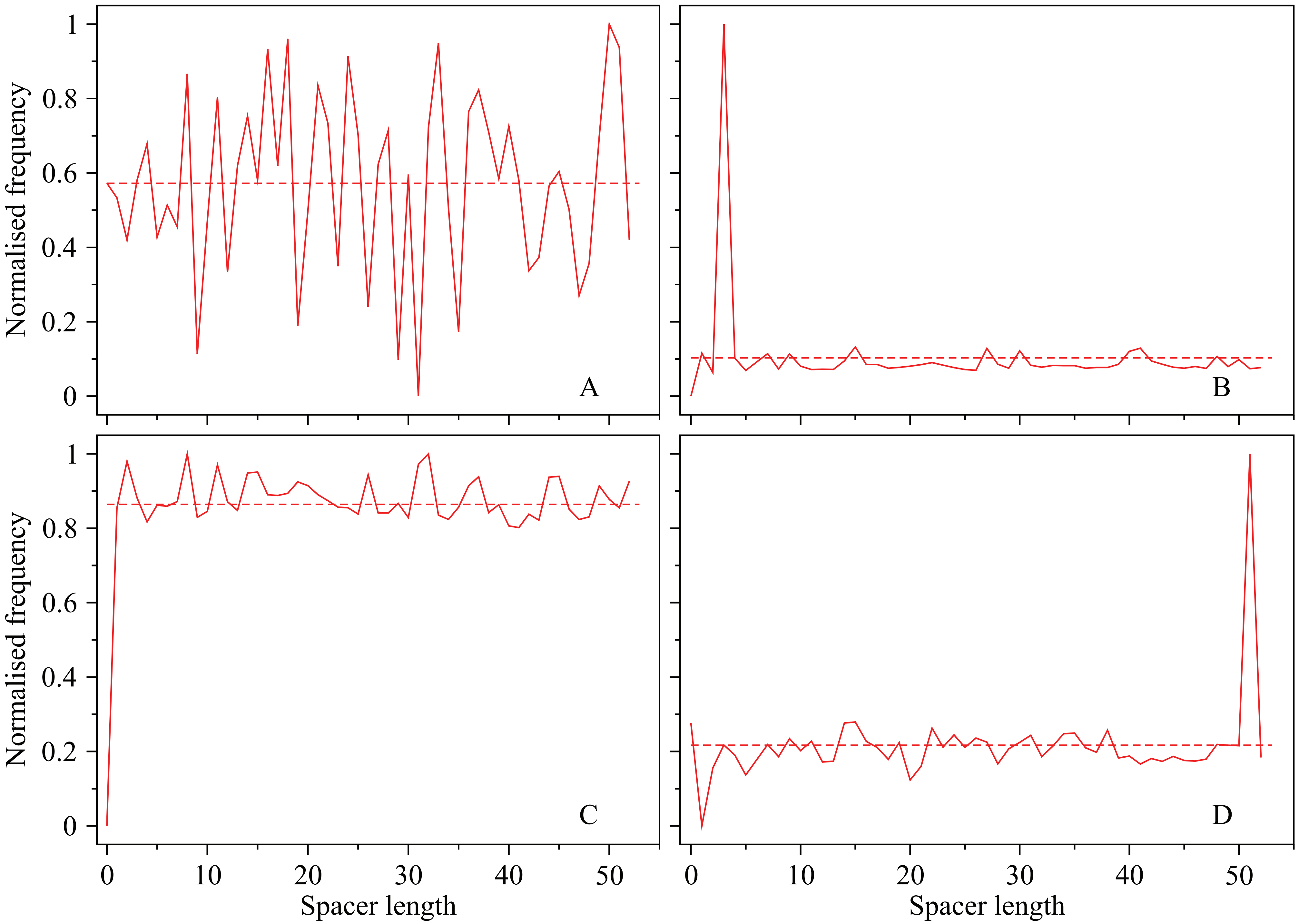

Normalised frequency
1
0.8
0.6
0.4
0.2
0
A
B
C
D
0
10
20
30
40
50
Spacer length

Fig. S3

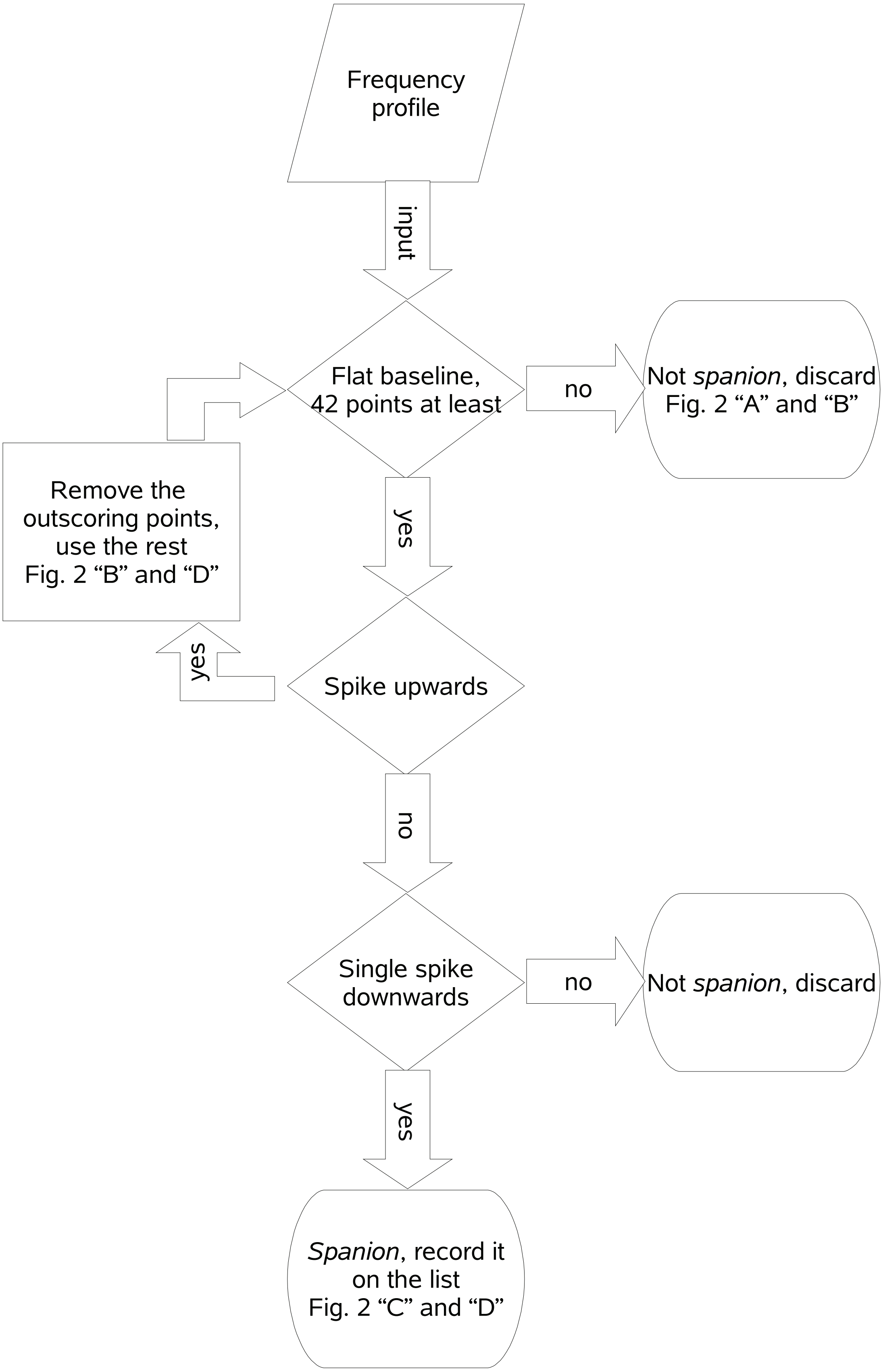
Frequency profile
input
Flat baseline, 42 points at least
no
Not *spanion*, discard Fig. 2 "A" and "B"
yes
Remove the outscoring points, use the rest Fig. 2 "B" and "D"
yes
Spike upwards
no
Single spike downwards
no
Not *spanion*, discard
yes
Spanion, record it on the list Fig. 2 "C" and "D"

Fig. S4

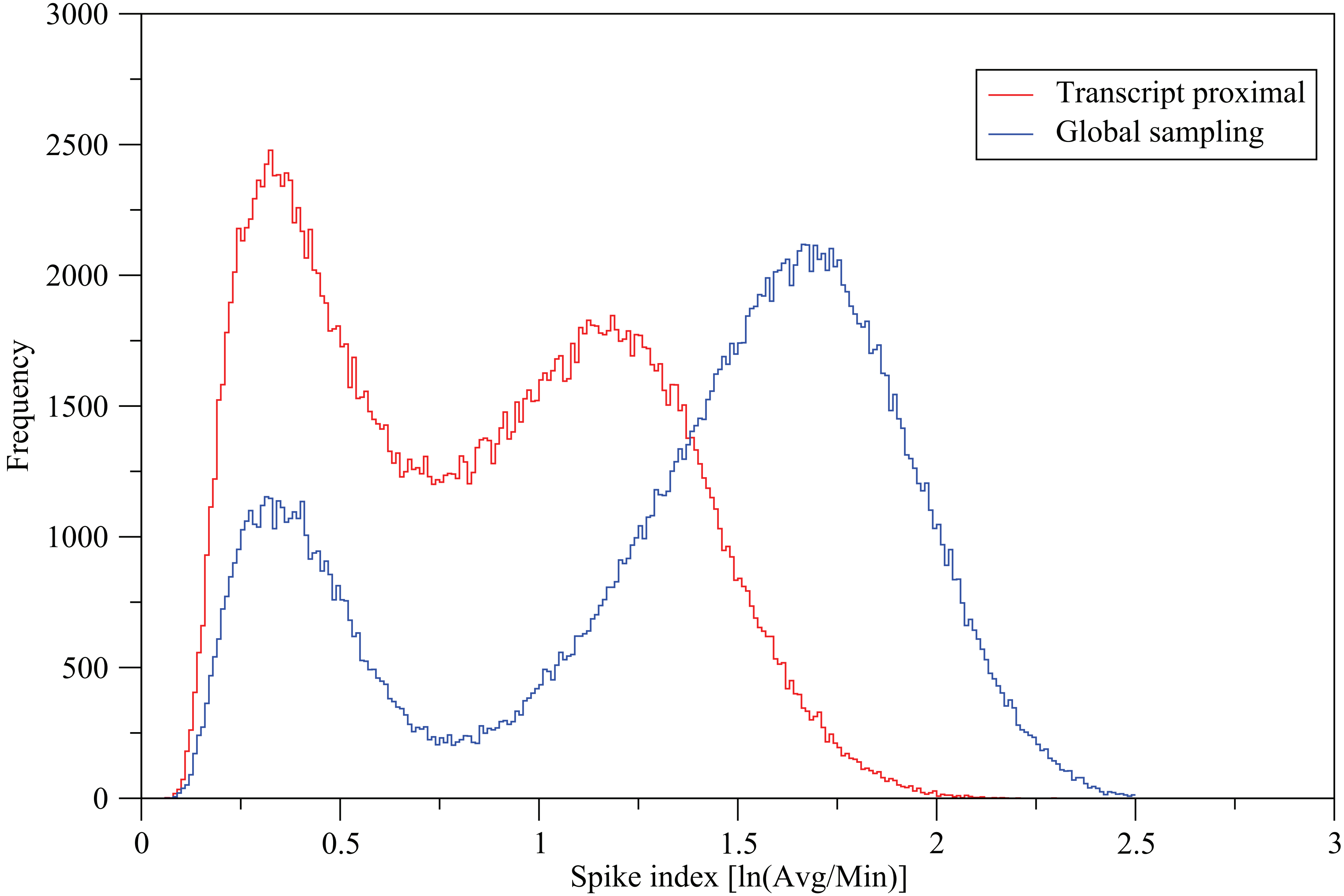

Transcript proximal
Global sampling
Frequency
Spike index [ln(Avg/Min)]
3000
2500
2000
1500
1000
500
0
0
0.5
1
1.5
2
2.5
3

Fig. S5

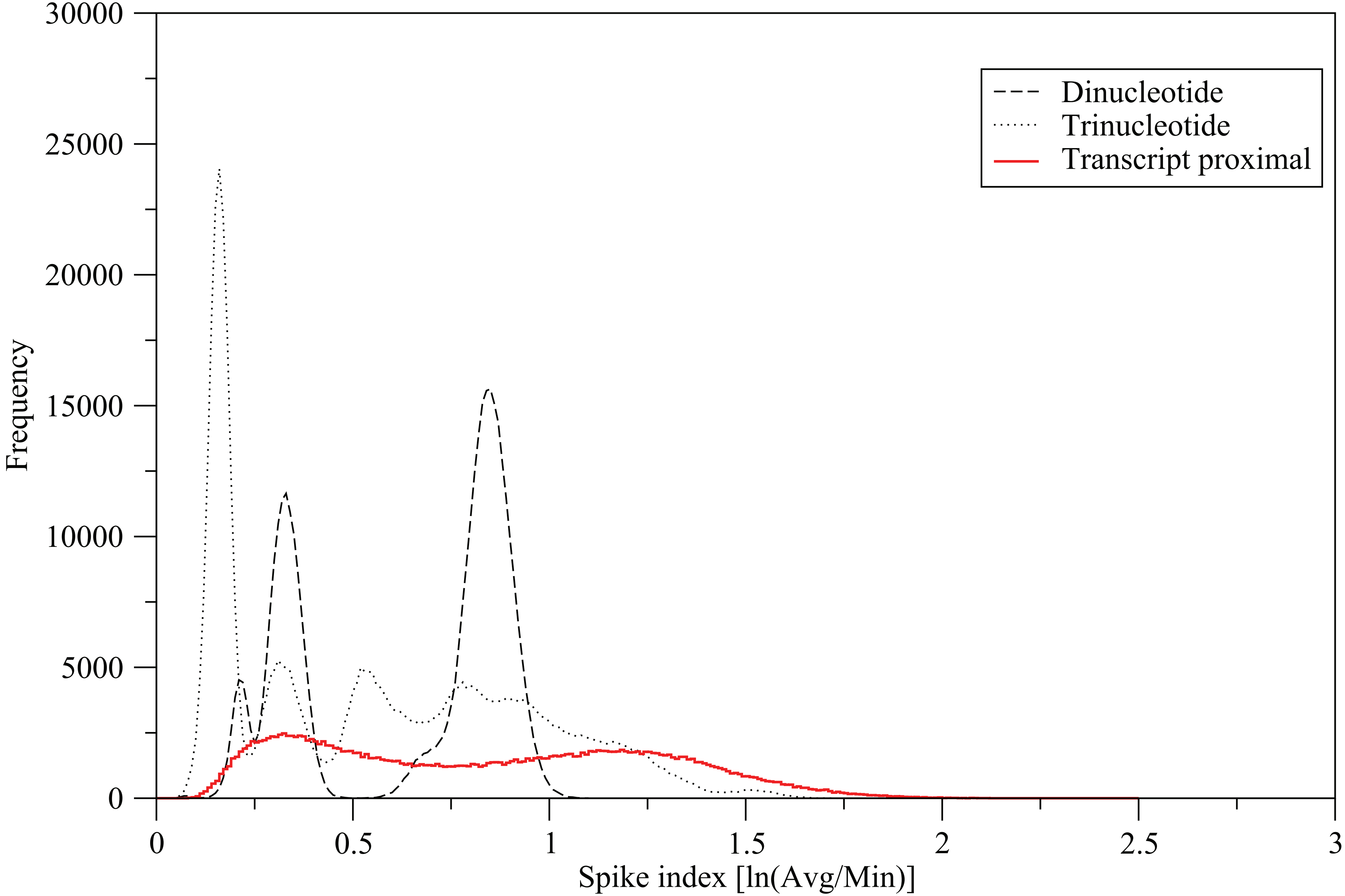
Dinucleotide
Trinucleotide
Transcript proximal
Frequency
Spike index [ln(Avg/Min)]
30000
25000
20000
15000
10000
5000
0
0
0.5
1
1.5
2
2.5
3

Fig. S6

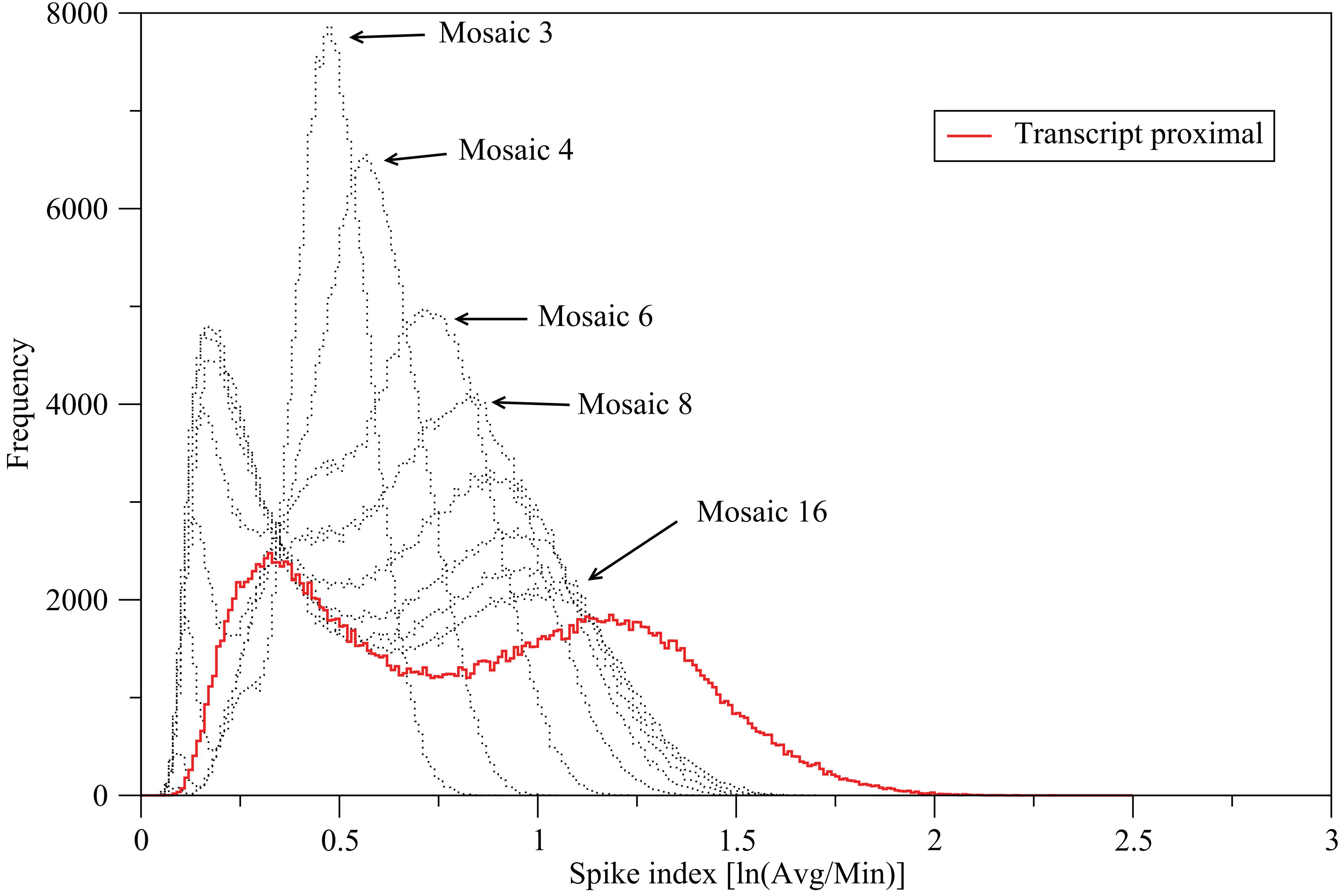

Mosaic 3
Mosaic 4
Mosaic 6
Mosaic 8
Mosaic 16
Transcript proximal
Frequency
Spike index [ln(Avg/Min)]
8000
6000
4000
2000
0
0
0.5
1
1.5
2
2.5
3

Fig. S7

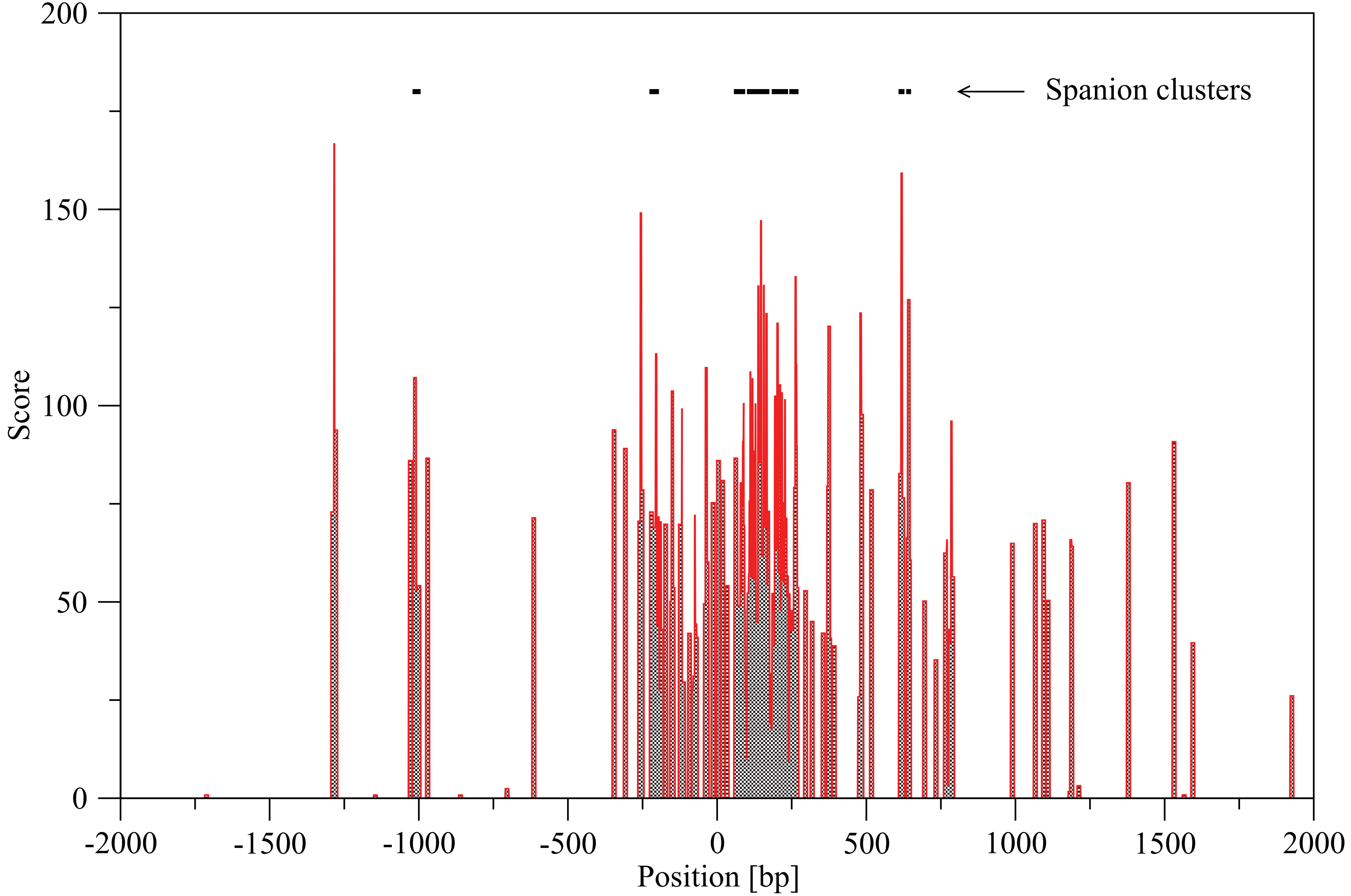
Spanion clusters
Score
Position [bp]
200
150
100
50
0
-2000
-1500
-1000
-500
0
500
1000
1500
2000

Fig. S8

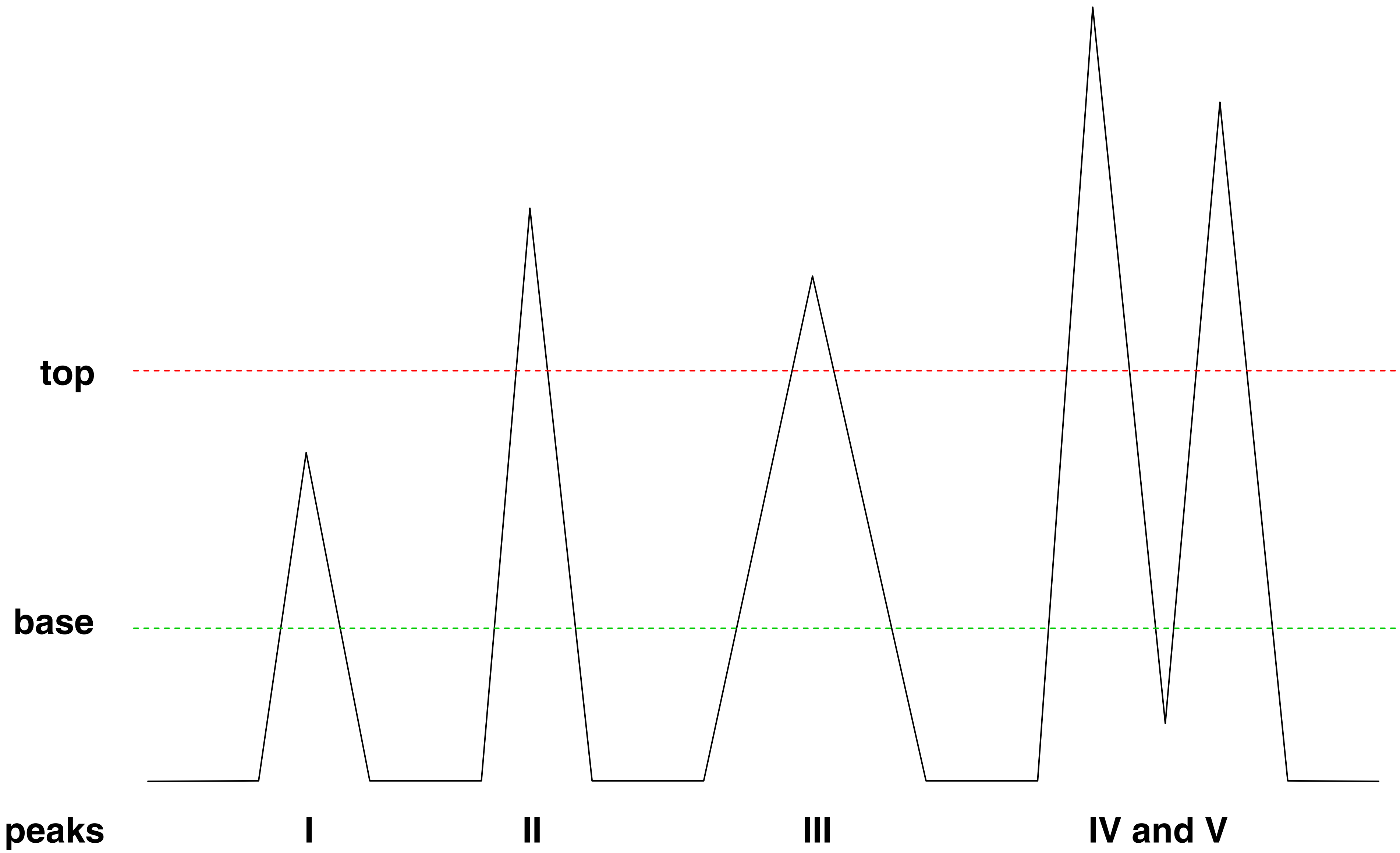

Fig. S9

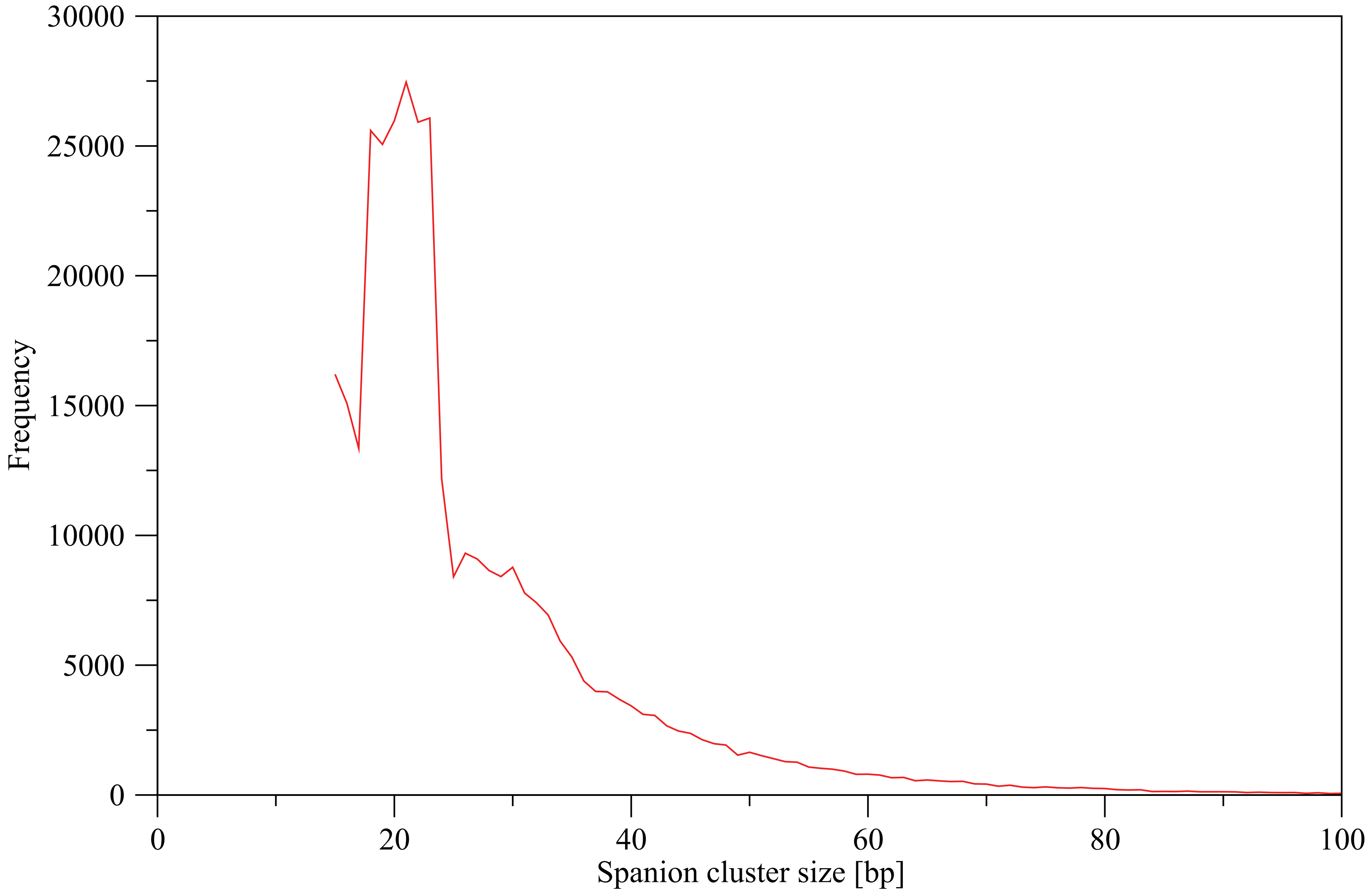
30000
25000
20000
15000
10000
5000
0
Frequency
0
20
40
60
80
100
Spanion cluster size [bp]
